# Enhancing children’s numeracy and executive functions via their explicit integration

**DOI:** 10.1038/s41539-025-00302-9

**Published:** 2025-02-18

**Authors:** Gaia Scerif, Jelena Sučević, Hannah Andrews, Emma Blakey, Sylvia U. Gattas, Amy Godfrey, Zachary Hawes, Steven J. Howard, Liberty Kent, Rebecca Merkley, Rosemary O’Connor, Fionnuala O’Reilly, Victoria Simms

**Affiliations:** 1 Department of Experimental Psychology, University of Oxford, Oxford, UK; 2 Department of Psychology, University of Sheffield, Sheffield, UK; 3 Department of Applied Psychology and Human Development, University of Toronto, Toronto, ON Canada; 4 Early Start and School of Education, University of Wollongong, Wollongong, NSW Australia; 5 Department of Cognitive Science, Carleton University, Ottawa, ON Canada; 6 School of Psychology, Ulster University, Coleraine, UK

**Keywords:** Psychology, Social sciences

## Abstract

Executive functions (EF) are crucial to regulating learning and are predictors of emerging mathematics. However, interventions that leverage EF to improve mathematics remain poorly understood. 193 four-year-olds (mean age = 3 years; 11 months pre-intervention; 111 female, 69% White) were assessed 5 months apart, with 103 children randomised to an integrated EF and mathematics intervention. Our pre-registered hypotheses proposed that the intervention would improve mathematics more than practice as usual. Multi-level modelling and network analyses were applied to the data. The intervention group improved more than the control group in overall numeracy, even when controlling for differences across settings in EF and mathematics-enhancing practices. EF and mathematics measures showed greater interconnectedness post-intervention. In addition, disadvantaged children in the intervention group made greater gains than in the control group. Our findings emphasise the need to consider EFs in their integration with co-developing functions, and in their educational and socio-economic context.

## Introduction

Executive functions (“EFs” henceforth) are cognitive skills that help maintain goals in mind, inhibit inappropriate responses and think flexibly. In adults, EFs form an overarching unitary construct that is also separable into distinct components, working memory updating (“WM”), inhibitory control (“IC”), and cognitive flexibility (“CF”)^1,2^. In younger participants, separable EF skills can be captured, but they cluster into a smaller number of latent factors, with one or two latent components underlying EF in preschool^3,4^. Multiple empirical and meta-analytic findings highlight robust and replicable correlations between mathematics and EF, both construed as a holistic construct and as separable components, throughout the primary school years^5–7^ and from the preschool period^8–15^. For example, a meta-analysis^10^ found that EF was modelled both as holistic and separate constructs related to mathematics achievement in preschool children. In addition, 3-year-olds’ holistic EF predicted mathematics achievement both later in the same year, and when they were five years of age^8^.

Despite this strong correlational and longitudinal evidence, interventions focused on training EF in isolation have failed to yield improvements in other correlated cognitive domains, including mathematics^16–18^. Recently, meta-analyses and position pieces have highlighted that interventions integrating EFs and mathematical content are more likely to improve early mathematics attainment, given that mathematics requires the integration of mathematics-specific skills and EF^19,20^. However, the empirical literature on integrated EF and mathematics interventions remains limited and requires further replication, particularly for very young children. A meta-analysis^19^ identified very few integrated interventions prior to school entry. Some studies report encouraging evidence of improvements in mathematics following combined mathematics and EF interventions^21–23^, but others report less success^24,25^ or failures to replicate^26^. Therefore, while integrating EF and co-developing functions like mathematics has the potential to improve mathematics, the empirical evidence, particularly in young children, remains limited and mixed.

Scerif and colleagues^20^ argued that these inconsistencies depend on a lack of explicit focus on the mechanisms of integration between EFs and mathematics. They hypothesise that for young children who are establishing their mathematical skills, practising EF challenge in the context of mathematical content provides opportunities for deeper processing and learning, thereby enhancing the co-development of EF and mathematics. This proposal focuses on testing the mechanisms of integrated interventions as a tool to improve mathematics achievement, but it is also consistent with broader and general theoretical frameworks for neurocognitive development, such as the interactive specialisation^27^ or mutualistic/transactional framework^6,13,28^, in which the dynamic interplay of domain-general control functions (such as EFs) and domain-specific skills (such as numerical cognition) promotes their change.

Indeed, large-scale longitudinal data suggest that EF does not act as a unidirectional influence on mathematics. Instead, these data support mutualistic influences between components of EF and mathematics^6,13^. For example, working memory updating and cognitive flexibility predict change in mathematical achievement, while at the same time, mathematical achievement predicts change in working memory updating and cognitive flexibility across the primary school years^6^ pointing to the co-development of EF and mathematics. This mutualistic interplay suggests that intervening in the integration of EF and mathematics would yield benefits to mathematics achievement in ways that, thus far, intervening on EF alone has failed to engender. The current study aims to test the efficacy of an integrated intervention, at a time when these skills are rapidly developing for very young children.

Early childhood is a period of great interest for integrated EF and mathematics interventions for theoretical and societal reasons. From a theoretical viewpoint, EF and mathematical skills are both developing rapidly and bidirectionally relationships from preschool, as detailed above^9,13,29^, so that integrated interventions may leverage this interplay. In addition, the preschool period offers a window of opportunity for societal uplift, as young children’s skills are rapidly emerging. Intervening at this juncture could lay the foundation with strong executive skills that may benefit their mathematical learning^30^, but also more broadly in how well they do at school and beyond. Indeed, there is evidence that EF and mathematics are not fixed skills, but are malleable, so that integrated interventions may benefit children growing up in conditions of disadvantage most because they have more to gain from such experiences^31^. However, the way in which integrated interventions improve outcomes for all children remains unclear and must be studied further.

Traditional analyses of the efficacy of intervention trials can fall short of studying how the interplay between multiple cognitive processes operates, because they focus on outcome variables in isolation (e.g., testing improvement in mathematics achievement, but not testing changes in contributing foundational mathematics, e.g., cardinality, or other skills, e.g., executive skills), instead of describing changes within networks of correlated cognitive skills. Recently, graph-theory-based network analyses have been championed to complement traditional univariate analyses, to characterise (changes in) the inter-relations between cognitive functions^32^. This novel approach is far less familiar in psychology than it is in other fields of science^33^, but it has strong potential value as a tool to investigate how integrated interventions operate and therefore better understand intervention-induced change across domains. In particular, network analysis enables researchers to consider how cognitive processes change in their inter-relationships following an integrated intervention, in a way that univariate statistics do not. For example, network analyses have been used to investigate changes in the interconnectedness of EF indices from before to after an EF-focused intervention in late childhood and adolescence^34^. This approach revealed multiple changes that extended beyond changes in univariate statistics following the intervention. More specifically, children’s EF network showed both weaker and fewer connections than the adolescent network prior to the intervention. However, post-intervention the children’s network had denser, more numerous and stronger connections, resembling the adolescent network. In addition, network analyses have been employed to test unitary models of EF and their component processes across the lifespan^35^. Most recently, network analytic approaches have found that distinct EFs (e.g., inhibition, working memory updating, cognitive flexibility) differ in their interconnectedness from childhood to adolescence^36^, reporting that inhibition is more densely interconnected and central to EF networks in childhood, whereas working memory updating takes this more central role in adolescence. To our knowledge, network models have not yet been used to model the inter-relations between EF and mathematics, either naturalistically, or following interventions that integrate EF and mathematics.

Therefore, there are a number of pressing limitations to the existing evidence base on integrated EF and mathematics interventions. First, the published evidence of integrated EF training remains limited, precluding meta-syntheses of results. This evidence is required to inform more explicit theories of change, and data on improvements in mathematics following integrated EF interventions are needed. Second, there remains a clear gap in understanding how the relationships across executive and mathematical skills change in the face of fast early development and integrated interventions. Network analyses can offer novel insights because they are able to supplement findings of quantitative improvements in individual cognitive skills, to additionally investigate changes in the relations among them.

The current study evaluated the efficacy of the Orchestrating Numeracy and the Executive (“ONE”) programme. This programme was designed to provide early childhood educators with training and supportive activities integrating EF and mathematics learning. The programme consisted of: (1) professional development (“PD”) for Early Years practitioners, focused on fostering educators’ understanding of EF in early mathematics, and (2) an induction into a set of 25 activities, co-developed with educators, predicated on integrating EF and early mathematics. The activities were designed to be easily embedded into preschool contexts and routines. The ONE followed the structure of a similarly paced PD-based intervention integrating EFs into play-based activities (albeit without a specific mathematics focus) in Australia (PRSIST^37^), which resulted in improvements in EF for the intervention settings (but not an improvement in mathematics attainment). The ONE adapted the delivery framework of PRSIST but combined the EF challenge with mathematics-specific content by generating new or modifying existing preschool activities. It aligned with the non-statutory Early Years curriculum guidance in the United Kingdom with the support and advice of UK-based Early Years Practitioners. The target mechanism of change was the explicit integration of the EF challenge embedded in mathematics activities. Here we evaluate mechanistic hypotheses about the effects of this integrated EF and mathematics intervention.

First, we hypothesised that early mathematics scores would improve to a greater degree for children in the intervention group than for a comparison group of children. This a priori hypothesis and the trial protocol were detailed in the Open Science Framework [https://osf.io/8y5u6/]. Our pre-registered hypotheses focused on testing improvements in mathematics achievement, as education intervention trials have tended to focus on this, and because previous cognitive studies of transfer effects from EF training have also used mathematics achievement as a target transfer variable. However, an improvement in EF itself is also an expected consequence of integrated mathematics and EF interventions. We, therefore, assessed improvements across a cumulative index of early mathematics (our primary outcome measure), and we also tested improvements in cumulative EF, as well as separate contributing numerical (e.g., counting, cardinality, ordinal processing) and EF (e.g., inhibitory control, cognitive flexibility, working memory) skills. Second, we used network-based approaches to test the hypothesis that the interconnectedness of EF and mathematics measures, as indexed by network parameters, changed more in the intervention group than in the comparison group.

## Results

### Intervention efficacy

Efficacy analyses focused on an intention-to-treat analytical approach. This conservative analytical approach treats children allocated to the intervention arm as having received the intervention, even if educators did not deliver activities to the requested level of adherence, either because this was not feasible, or because of other constraints. We report information on the feasibility, acceptability, adherence and implementation quality of the programme in the supplementary materials.

#### Mathematics

**Fig. 1:**
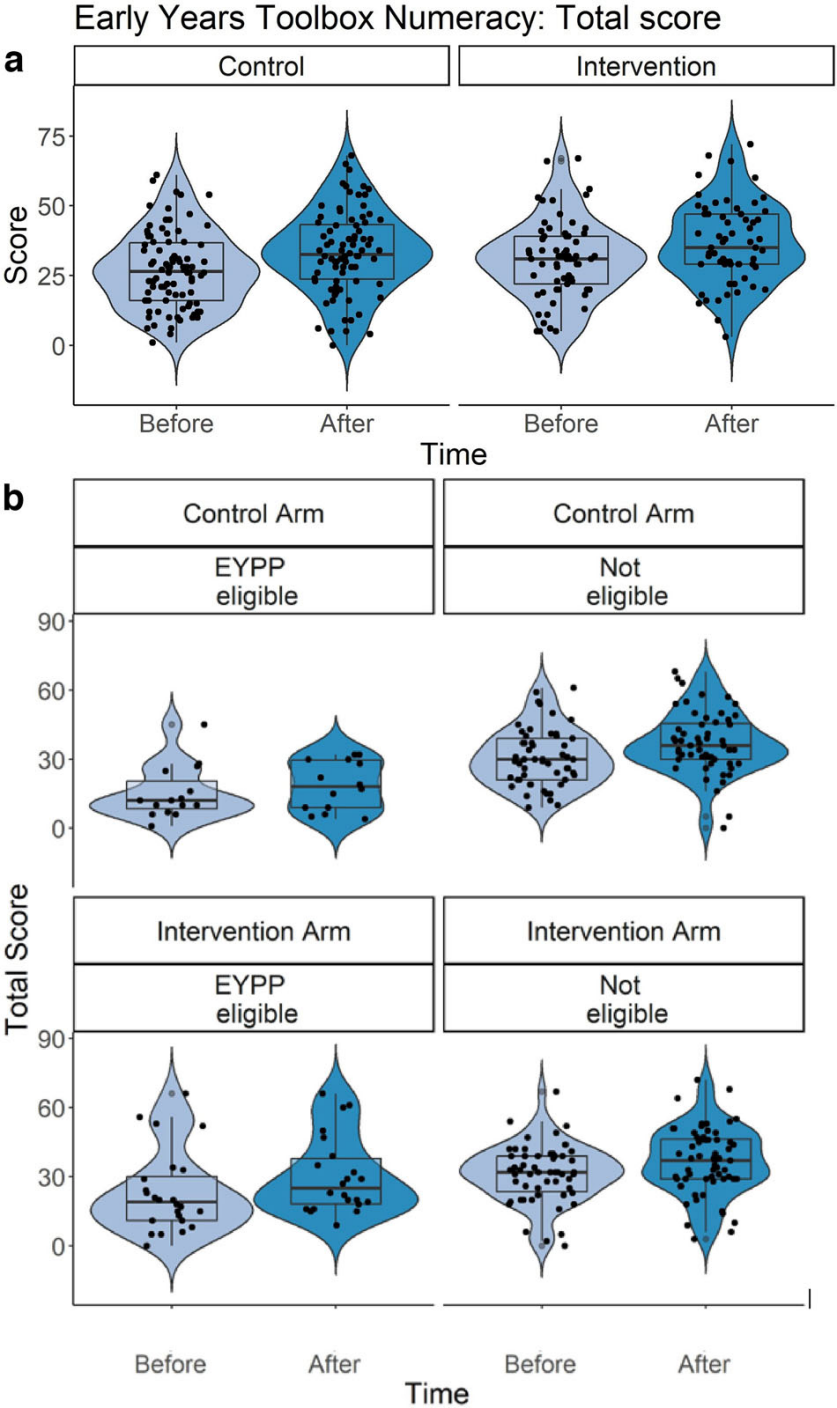
Statistically significant main effect of Intervention and Intervention * EYPP eligibility Interaction Effect for Numeracy Scores. Numeracy scores (EYTN) in the control and intervention group, split by assessment time (before and after the intervention) first presented together (**a**) and then split into EYPP eligible and children not eligible for EYPP (**b**). Box plots depict the median, median, minimum, maximum and interquartile range, and superimposed violin plot showing distribution of the data. Black dots represent individual children. In (**b**), the vertical dimension represents the comparison between control and intervention arm, on similarly scaled axes.

Unadjusted means, estimated marginal means (with standard deviations), statistics (F, p) and effect sizes (Hedge’s g) for all mathematics measures are reported in Table 1. For EYTN, there was a statistically significant main effect of the Intervention group, driven by higher improvements in numeracy for children in the Intervention group compared to children in the Control group (see Fig. 1a). There were also main effects of the intervention on Give N and Number Comparison, again driven by higher improvements in the Intervention compared to the Control Group. EYPP eligibility had a significant main effect on all mathematics variables except for Count High and Order Processing. For all main effects, children who grew up at a disadvantage (eligible for EYPP) had significantly lower scores compared to non-EYPP-eligible children.

**Table 1:** Effects of intervention, disadvantage and their interaction on mathematics variables

*Measure*	*Group*	*Time 1 Unadj. Mean (stdev)*	*Time 2 Unadj. Mean (stdev)*	*Effect*	*Estimated Marginal Means (stdev)*	*F ratio*	*P*	*Hedge’s g*
*Early Years Toolbox Numeracy(raw score)*	***Intervent****.*	28.59 (13.88)	34.91 (14.90)	***Intervention***	*M*_Con_ = 25.99 (14.58); *M*_Int_ = 32.18 (14.70)	**F(1,21)** = **7.44**	***0.012***	***0.42***
	***Control***	27.07 (13.69)	33.43 (14.83)	***EYPP***	*M*_EYPP_Yes_ = 22.61 (16.21); *M*_EYPP_No_ = 33.47(13.26)	**F(2,210)** = **15.08**	***<0.001***	***0.77***
				Int*EYPP	*M*_EYPP_Yes_Con_ = 16.65 (11.32); *M*_EYPP_Yes_Int_ = 28.56 (17.42)	**F(2,210)** = **3.38**	***0.036***	***0.64***
*Count High (maximum count)*	***Intervent****.*	16.84 (18.31)	23.25 (23.17)	***Intervention***	*M*_Con_ = 15.53 (14.44); *M*_Int_ = 19.29 (21.09)	F(1,25) = 2.00	0.170	
	***Control***	15.57 (17.67)	17.91 (9.78)	***EYPP***	*M*_EYPP_Yes_ = 13.66 (20.24); *M*_EYPP_No_ = 19.44 (17.51)	F(2,164) = 2.70	0.070	
				Int*EYPP	*M*_EYPP_Yes_Con_ = 11.70 (17.25); *M*_EYPP_Yes_Int_ = 15.63 (19.77)	F(2,161) = .03	0.967	
*Give N (score)*	***Intervent****.*	6.47 (4.66)	8.77 (4.76)	***Intervention***	*M*_Con_ = 5.83 (4.79); *M*_Int_ = 8.60 (4.80)	**F(1,27)** = **11.25**	***0.002***	***0.58***
	***Control***	8.00 (4.84)	8.36 (4.76)	***EYPP***	*M*_EYPP_Yes_ = 4.65 (4.93); *M*_EYPP_No_ = 8.75 (9.17)	**F(2,282)** = **20.63**	***<0.001***	***0.49***
				Int*EYPP	*M*_EYPP_Yes_Con_ = 2.68 (4.61); *M*_EYPP_Yes_Int_ = 6.61 (4.86)	F(2,286) = 1.07	0.343	
*Number Comparison (proportion correct)*	***Intervent****.*	0.57 (0.19)	0.66 (0.19)	***Intervention***	*M*_Con_ = 0.55 (0.21); *M*_Int_ = 0.62 (0.19)	**F(1,32)** = **4.58**	***0.040***	***0.35***
	***Control***	0.54 (0.22)	0.63 (0.19)	***EYPP***	*M*_EYPP_Yes_ = 0.55 (0.19); *M*_EYPP_No_ = 0.63 (0.20)	**F(2,216)** = **4.20**	***0.016***	***0.40***
				Int*EYPP	*M*_EYPP_Yes_Con_ = 0.48 (0.11); *M*_EYPP_Yes_Int_ = 0.61 (0.19)	F(2,219) = 1.32	0.270	
*Number Naming (score)*	***Intervent****.*	11.22 (6.52)	13.00 (5.25)	***Intervention***	*M*_Con_ = 11.47 (5.87); *M*_Int_ = 11.53 (5.97)	F(1,38) = .003	0.956	
	***Control***	11.79 (6.29)	13.59 (5.37)	***EYPP***	*M*_EYPP_Yes_ = 9.56 (7.11); *M*_EYPP_No_ = 13.27 (4.98)	**F(2,276)** = **9.76**	***<0.001***	***0.66***
				Int*EYPP	*M*_EYPP_Yes_Con_ = 8.49 (6.79); *M*_EYPP_Yes_Int_ = 10.63 (7.16)	F(2,278) = 1.98	0.140	
*Order Processing (score)*	***Intervent****.*	1.15 (2.84)	2.97 (4.21)	***Intervention***	*M*_Con_ = 1.69 (3.78); *M*_Int_ = 1.98 (3.68)	F(1,27) = .27	0.610	
	***Control***	1.75 (3.64)	2.54 (3.92)	***EYPP***	*M*_EYPP_Yes_ = 1.44 (3.39); *M*_EYPP_No_ = 2.40 (3.38)	F(2,187) = 1.92	0.149	
				Int*EYPP	*M*_EYPP_Yes_Con_ = 0.65 (1.12); *M*_EYPP_Yes_Int_ = 2.23 (3.93)	F(2,190) = 1.75	0.177	
*BAS – PC (t-score)*	***Intervent****.*	51.91 (11.41)	53.99 (10.72)	***Intervention***	*M*_Con_ = 50.29 (11.71); *M*_Int_ = 53.26 (11.09)	F(1,27) = 2.99	0.095	
	***Control***	51.55 (12.68)	53.98 (10.58)	***EYPP***	*M*_EYPP_Yes_ = 47.27 (13.07); *M*_EYPP_No_ = 54.24 (10.38)	**F(2,203)** = **10.23**	***<0.001***	***0.63***
				Int*EYPP	*M*_EYPP_Yes_Con_ = 43.31 (10.19); *M*_EYPP_Yes_Int_ = 51.23 (13.15)	**F(2,206)** = **4.26**	***0.015***	***0.65***

Please note that condition means and effect size indices for the main effect of EYPP are reported only for children whose status was confirmed, and for the interaction effect, they are reported for disadvantaged children (EYPP eligible) here, for brevity. Effect size (Hedge’s *g*) is also reported for statistically significant effects only, for brevity. Other estimated marginal means are reported in the Supplementary Online Materials (Supplementary Table 2).

*EYPP* Eligible for Early Years Pupil Premium, a UK-based index of economic disadvantage, *EYPP_Yes* EYPP Eligible, therefore disadvantaged, *Con* Control, *Int* Intervention, Int*EYPP Interaction effect between Intervention and EYPP eligibility, *BAS – PC* British Ability Scale, Pattern Construction.

Bold and italic values highlight statistically significant effects, associated *p* values and Hedge's *g.*

**Fig. 2:**
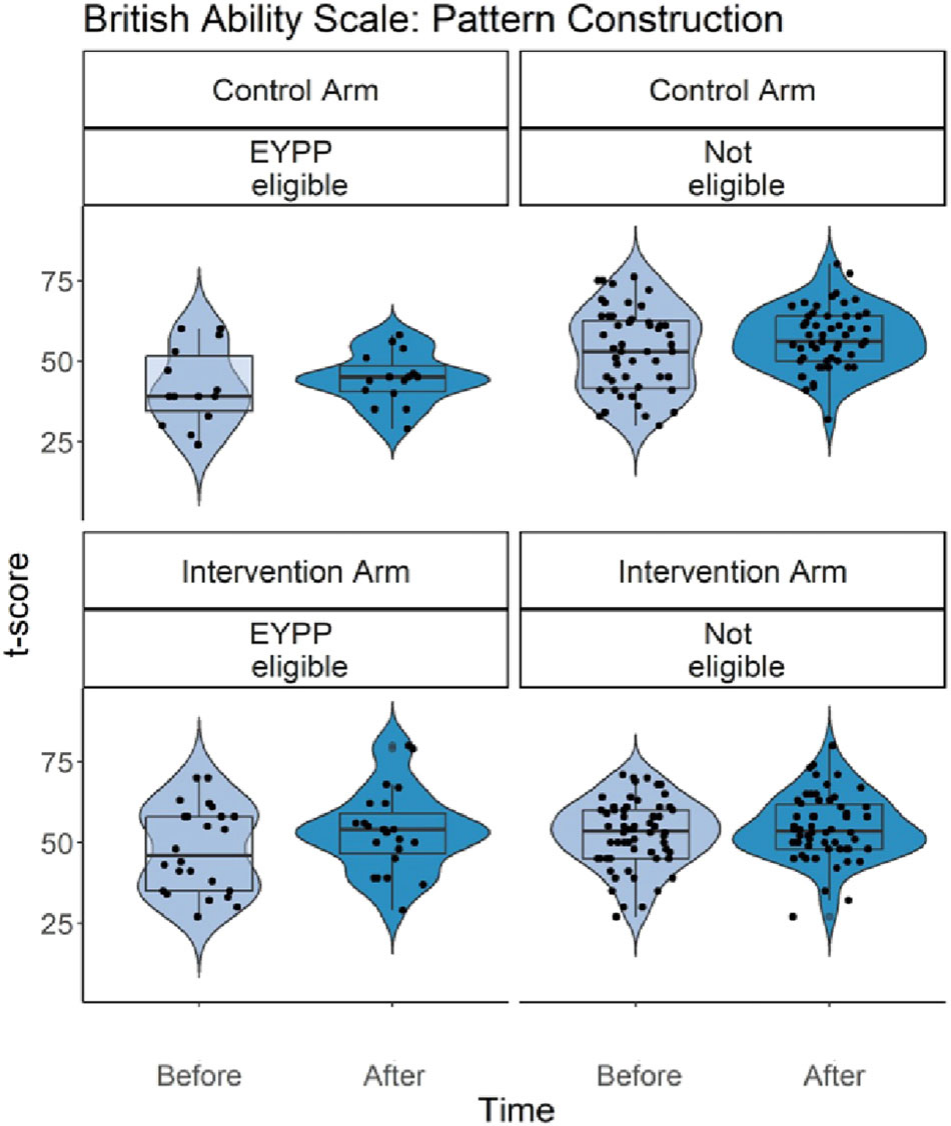
Statistically significant effects for British Ability Scale - Pattern Construction scores. British Ability Scale - Pattern Construction (indexing spatial ability) in the control and intervention group, split by assessment time (before and after the intervention) and into EYPP eligible and children not eligible for EYPP. Box plots depict the median, median, minimum, maximum and interquartile range, and superimposed violin plots showing the distribution of the data. Black dots represent individual children. The vertical dimension represents the comparison between the control and intervention arms, on similarly scaled axes.

In addition, for EYTN, there was also a statistically significant Intervention * EYPP interaction effect (see Fig. 1b). For children with EYPP eligibility, changes in EYTN scores were larger in the Intervention group (T1 = 25.04; T2 = 32.07) than in the Control group (T1 = 14.86, T2 = 18.48, *p* = 0.001). In addition, EYPP-eligible children scored less well on this overall numeracy measure than non-eligible children in the Control group (*p* < 0.001), but this difference was reduced for children in the Intervention group (*p* = 0.026). Furthermore, for spatial skills (as indexed by BAS3-PC), there was also an Intervention * EYPP eligibility interaction effect (see Fig. 2). Children with EYPP eligibility improved more in the Intervention group (T1 = 48.49; T2 = 53.98) than in the Control group (T1 = 42.31; T2 = 44.30). Children with EYPP eligibility had poorer spatial skills than children without EYPP eligibility in the Control group, *p* < 0.001, but not in the intervention group, *p* = 0.366. In addition, children with EYPP in the Intervention arm had better spatial skills than children with EYPP in the control group, *p* = 0.006. None of the other main or interaction effects reached statistical significance.

#### Executive functions

**Fig. 3:**
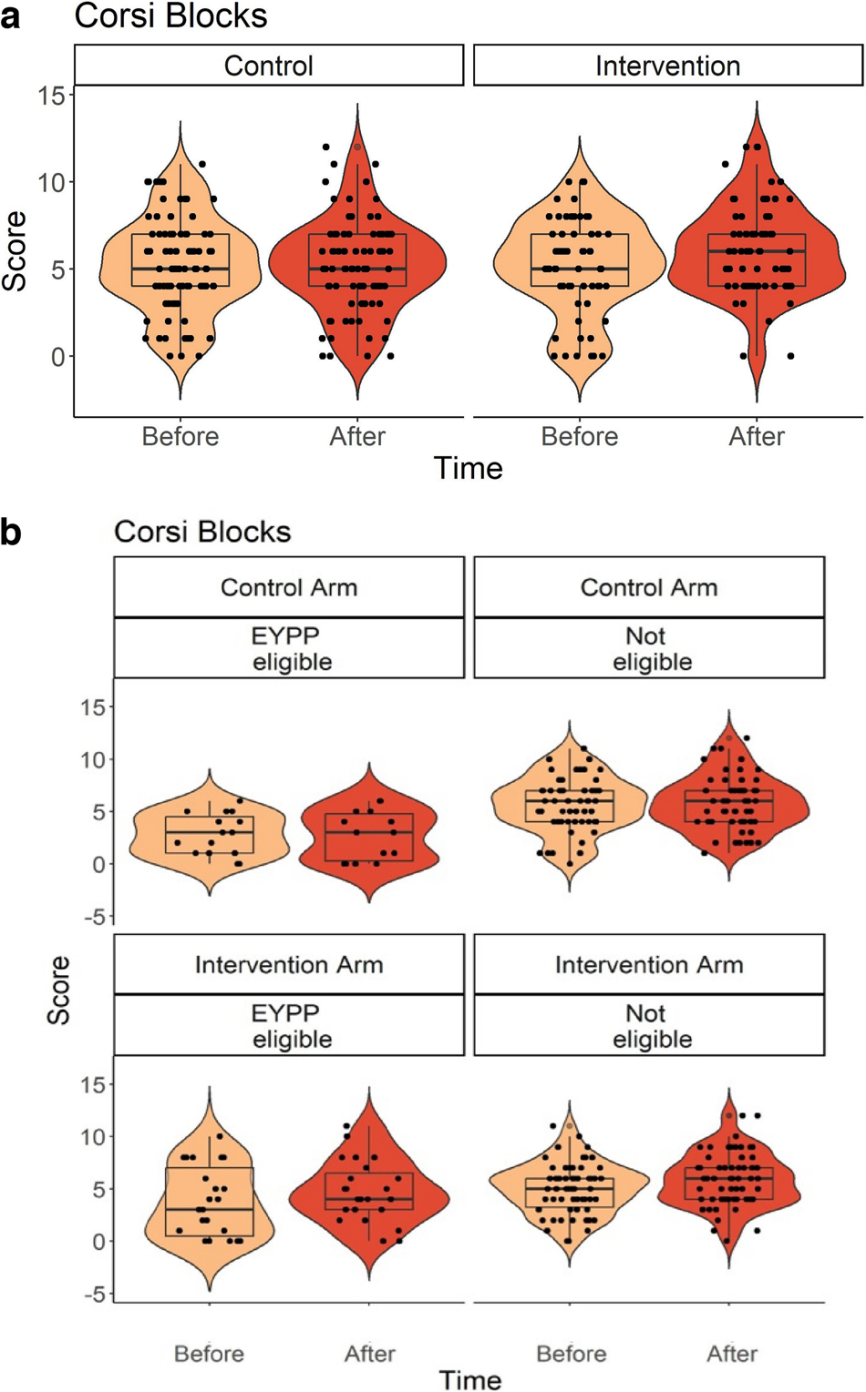
Statistically Significant Effects for Corsi Block scores. Statistically significant Main Intervention Effect and Intervention * EYPP Interaction Effect. Corsi Block scores (indexing maintenance in working memory) in the control and intervention group, split by assessment time (before and after the intervention) first presented together (**a**) and then split into EYPP eligible and children not eligible for EYPP (**b**). Box plots depict the median, median, minimum, maximum and interquartile range, and superimposed violin plots showing the distribution of the data. Black dots represent individual children. In (**b**), the vertical dimension represents the comparison between the control and intervention arms, on similarly scaled axes.

The unadjusted condition means, estimated marginal means (with standard deviation), statistics (F, p) and effect sizes (Hedge’s g) for EFs measures are reported in Table 2. There was a main effect of the intervention on the Corsi Blocks Score (see Fig. 3a), but there were no other statistically significant main intervention effects. EYPP eligibility had a significant main effect on all EF variables, except for Rabbits and Boats. For all main effects, children who grew up at a disadvantage (eligible for EYPP) had significantly lower scores compared to EYPP-not-eligible children and children whose status was unknown.

**Table 2:** Effects of intervention, economic disadvantage and their interaction on EF variables

*Measure*	*Group*	Time 1 Unadj. Mean (stdev)	Time 2 Unadj. Mean (stdev)	*Effect*	*Estimated Marginal Means (stdev)*	*F ratio*	*P*	*Hedge’s g*
*Corsi Blocks (score)*	***Intervention***	4.84 (2.80)	5.60 (2.79)	***Intervention***	*M*_Con_ = 4.48 (2.64); *M*_Int_ = 5.34 (2.81)	**F(1,28)** = **4.55**	***0.042***	***0.31***
	***Control***	5.18 (2.65)	5.17 (2.64)	***EYPP***	*M*_EYPP_Yes_ = 3.40 (2.95); *M*_EYPP_No_ = 5.55 (2.55)	**F(2,224)** = **19.23**	***<0.001***	***0.80***
				Int*EYPP	*M*_EYPP_Yes_Con_ = 2.60 (2.13); *M*_EYPP_Yes_Int_ = 4.19 (3.15)	**F(2,225)** = **3.87**	***0.022***	***0.56***
*Mr Ant (score)*	***Intervention***	1.29 (0.70)	1.48 (0.82)	***Intervention***	*M*_Con_ = 1.28 (0.77); *M*_Int_ = 1.38 (0.77)	F(1,24) = .56	0.460	
	***Control***	1.35 (0.76)	1.55 (0.77)	***EYPP***	*M*_EYPP_Yes_ = 1.16 (0.78); *M*_EYPP_No_ = 1.48 (0.75)	**F(2,232)** = **4.18**	***0.016***	***0.42***
				Int*EYPP	*M*_EYPP_Yes_Con_ = 0.90 (0.76); *M*_EYPP_Yes_Int_ = 1.42 (0.78)	**F(2,236)** = **4.51**	***0.012***	***0.66***
*Rabbits & Boats (post-switch score)*	***Intervention***	3.36 (4.17)	5.26 (4.26)	***Intervention***	*M*_Con_ = 4.57 (4.29); *M*_Int_ = 4.54 (4.31)	F(1,28) = 0.001	0.973	
	***Control***	4.47 (4.22)	5.64 (4.30)	***EYPP***	*M*_EYPP_Yes_ = 4.11 (4.28); *M*_EYPP_No_ = 5.00 (4.32)	F(2,267) = 1.08	0.340	
				Int*EYPP	*M*_EYPP_Yes_Con_ = 4.22 (4.06); *M*_EYPP_Yes_Int_ = 4.01 (4.21)	F(2,269) = 0.164	0.849	
*Go-no-go (impulse control score)*	***Intervention***	0.49 (0.20)	0.59 (0.20)	***Intervention***	*M*_Con_ = 0.54 (0.20); *M*_Int_ = 0.54 (0.21)	F(1,24) = 0.03	0.863	
	***Control***	0.51 (0.19)	0.59 (0.21)	***EYPP***	*M*_EYPP_Yes_ = 0.49(0.22); *M*_EYPP_No_ = 0.56(0.21)	**F(2,153)** = **3.96**	***0.021***	***0.33***
				Int*EYPP	ME_EYPP_Yes_Con_ = 0.48 (0.20); *M*_EYPP_Yes_Int_ = 0.49 (0.21)	F(2,153) = 0.09	0.918	
*EF latent factor (factor scores)*	***Intervention***	−0.09 (0.99)	0.004 (0.97)	***Intervention***	*M*_Con_ = −0.21 (0.10); *M*_Int_ = −0.01 (0.98)	F(1,26) = 1.88	0.183	
	***Control***	0.10 (1.00)	−0.004 (0.99)	***EYPP***	*M*_EYPP_Yes_ = −0.56(1.01); *M*_EYPP_No_ = 0.131(0.97)	**F(2,245)** = **14.03**	***<0.001***	***0.70***
				Int*EYPP	ME_EYPP_Yes_Con_ = −0.84 (0.93); *M*_EYPP_Yes_Int_ = −0.27 (1.04)	**F(2,245)** = **3.22**	***0.042***	***0.57***

Please note that condition means and effect size indices for the main effect of EYPP are reported only for children whose status was confirmed, and for the interaction effect, they are reported for disadvantaged children (EYPP eligible) here, for brevity. Effect size (Hedge’s *g*) is also reported for statistically significant effects only, for brevity. Other estimated marginal means are reported in the Supplementary Online Materials (Supplementary Table 3).

*EYPP* Eligible for Early Years Pupil Premium, a UK-based index of economic disadvantage, Int*EYPP Interaction effect for Intervention and EYPP status, *Con* Control, *Int* Intervention.

Bold and italic values highlight statistically significant effects, associated *p* values and Hedge's *g.*

**Fig. 4:**
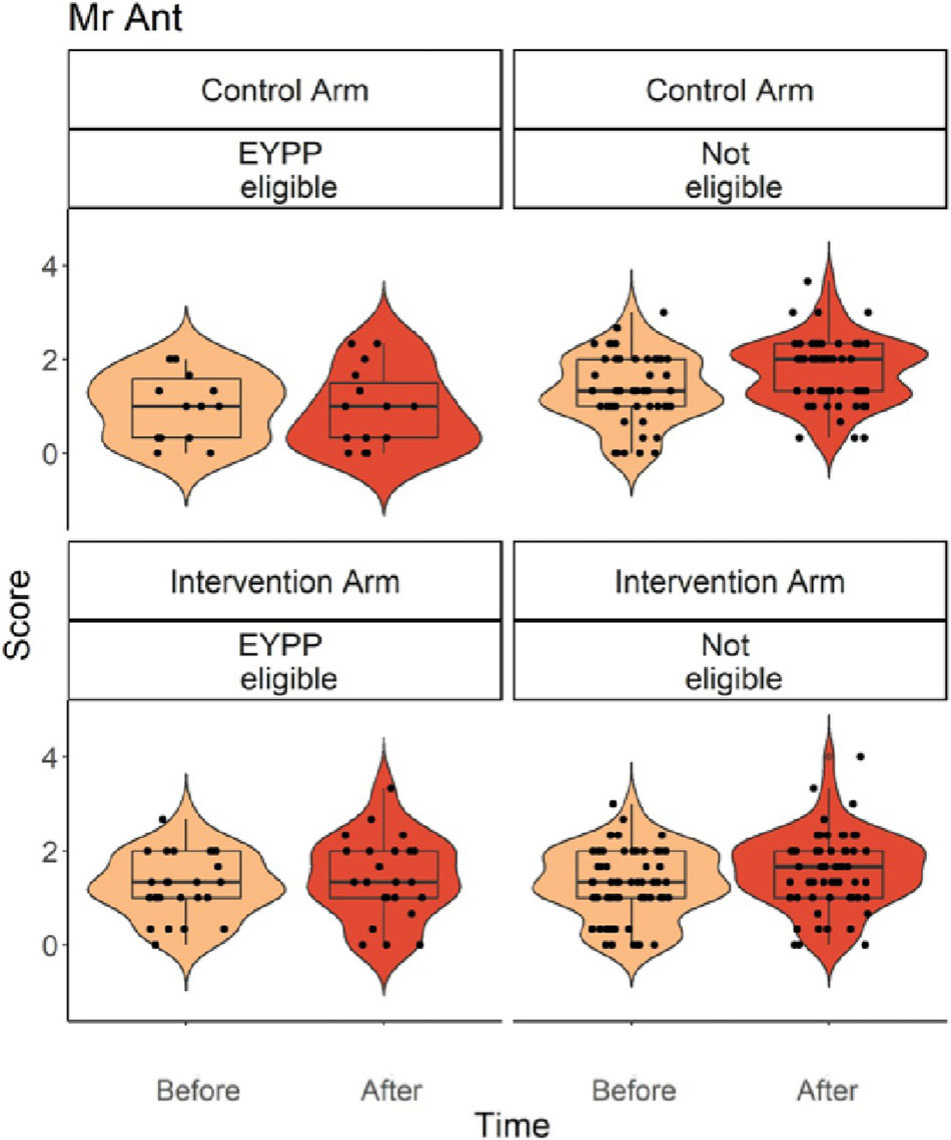
Statistically Significant Intervention * EYPP eligibility Interaction Effect for Mr Ant. Mr Ant scores (indexing maintenance in working memory) in EYPP eligible and children not eligible for EYPP, in the control and intervention group, split by assessment time (pre- and post-intervention). Box plots depict the median, median, minimum, maximum and interquartile range, and superimposed violin plots showing the distribution of the data. Black dots represent individual children. The vertical dimension represents the comparison between the control and intervention arms, on similarly scaled axes.

In addition, there was a significant Intervention * EYPP eligibility interaction effect for Corsi Blocks, for Mr Ant and for the EF latent variable (Figs. 3b and 4). For Corsi Blocks, the interaction effect was driven by significantly greater changes in Corsi Blocks scores for children who were EYPP eligible in the Intervention Group (T1 = 3.68; T2 = 4.70) compared to EYPP-eligible children in the Control group (T1 = 2.58; T2 = 2.63, *p* = 0.020). EYPP-eligible children in the Control group scored lower than non-EYPP-eligible children (*p* < 0.001), but this difference was smaller for EYPP-eligible children in the Intervention group (*p* = 0.011). For Mr Ant, the interaction effect was again driven by a greater change in scores for EYPP children in the Intervention group (T1 = 1.35; T2 = 1.50) compared to the Control group, (T1 = 91; T2 = 93, *p* = 0.009). In addition, EYPP-eligible children scored less well than EYPP non-eligible children in the Control Group (*p* < 0.001), but not in the intervention group (*p* = 0.932). For the latent EF variable, the interaction effect was driven by significantly greater change factor scores for children who were EYPP eligible in the Intervention Group (T1 = −0.38; T2 = −0.17), compared to EYPP-eligible children in the Control group, (T1 = −0.78; T2 = −0.90, *p* = 0.021). EYPP-eligible children in the Control group had lower EF factor scores than non-EYPP-eligible children (*p* < 0.001), but this difference was not significant for EYPP-eligible children in the Intervention group (*p* = 0.170). None of the other main or interaction effects reached statistical significance.

### Intervention mechanisms: network analyses

**Fig. 5:**
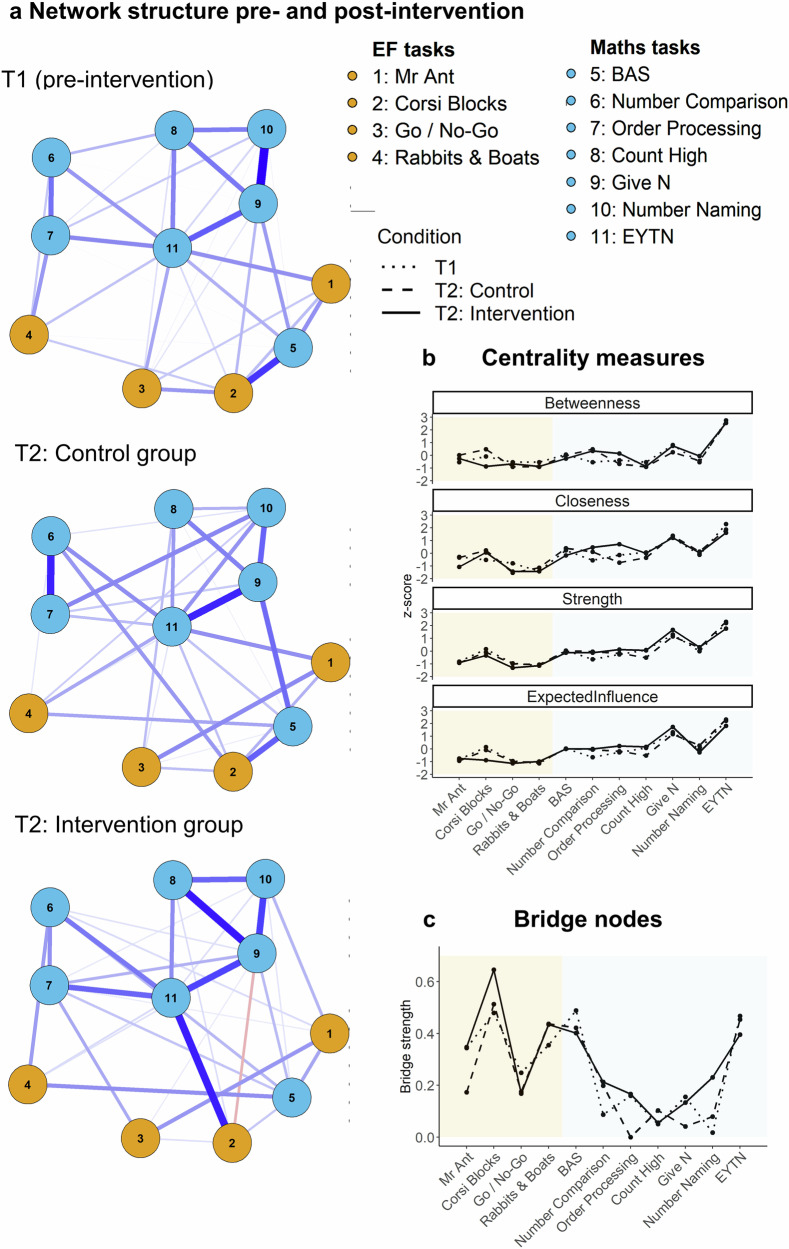
The EF-mathematics network model. The EF-mathematics network model. **a** Network structure for the overall sample prior to the intervention (Time 1) and in the control and the intervention group (Time 2). **b** Centrality indices for the three estimated networks: the Strength index refers to the absolute sum of all edges to a particular node, the Expected Influence index takes into account an edge sign (positive or negative), Betweenness refers to how often a node is on the shortest path between other nodes, Closeness refers to a mean distance from a node to all other nodes in the network. Values on the y-axis represent the standardised centrality coefficients (z-scores) for each centrality measure. The x-axis depicts the network nodes. **c** Bridge nodes (nodes in one domain most strongly connected to all nodes from the other domain): Corsi Blocks and EYTN are the strongest bridge nodes across networks. The first four nodes represent EF tasks (orange background) and the later 7 nodes represent mathematics tasks (blue background).

The above analysis revealed that the intervention led to improvements in a number of individual mathematics and EF indices. To better understand how the intervention impacted the relations between EF and maths skills, we complemented these univariate analyses with network analysis. The network analysis revealed that EF and mathematics are highly connected. In addition, the structure and strength of edges differed in the T2 intervention network compared to the T2 control network (see Fig. 5, and Supplementary Fig. 3 for bivariate correlations across all variables). The T1 network included both sets of children who were later randomised to either control or intervention settings. This is because the smallest network at the first time point (the network for control children, *N* = 90) failed to converge, likely because of the small sample and because children at Time 1 were younger and more variable in performance than they all became 5 months later. We therefore refer to the network prior to the intervention as the T1 overall network throughout. The T2 control network was more similar to the T1 overall network than the T2 intervention network, as indicated by their correlation coefficients (T2 control network correlation with T1 overall network: *r* = 0.714; T2 intervention network correlation T1 overall network: *r* = 0.566), showing that the intervention network differed from Time 1 more than the control network.

A focus on additional network parameters gave further insights into the ways in which this difference operated (see Fig. 5). First, nodes in the T2 intervention network showed increased centrality (i.e., increased connectedness), as indexed by higher Strength, Expected Influence, Closeness, and Betweenness, of several mathematics nodes (Fig. 5b). For example, there was greater connectedness for Number Comparison and Order Processing after the intervention, measures that require high integration of EF and mathematical knowledge compared to other measures (e.g., Number Naming or Count High, which rely on rote learning). In turn, these differences supported the view that integrated EF and mathematics activities strengthen the connectedness of these skills. Second, the strength and connections of bridge nodes between EF and mathematics clusters differed in the T2 intervention network compared to the T2 control network. For example, Corsi Blocks (an index of maintenance in memory) was identified as the main EF bridge node, and the EF node that was most strongly connected to all mathematics nodes (Fig. 5c). Corsi Blocks was most strongly connected to BAS – PC (an index of spatial skills) in the T1 overall network (*r* = 0.307) and T2 control network (*r* = 0.251), but it was most strongly connected to EYTN (an index of overall numeracy) in the T2 intervention network (*r* = 0.335). Furthermore, the strength of the Corsi Blocks bridge node was higher in the T2 intervention group than in the T2 control group, suggesting that the intervention might have contributed to the integration, increasing the impact of EF nodes on mathematics nodes. In turn, this added support to the suggestion that integrated approaches support the co-development of EF and mathematics.

**Fig. 6:**
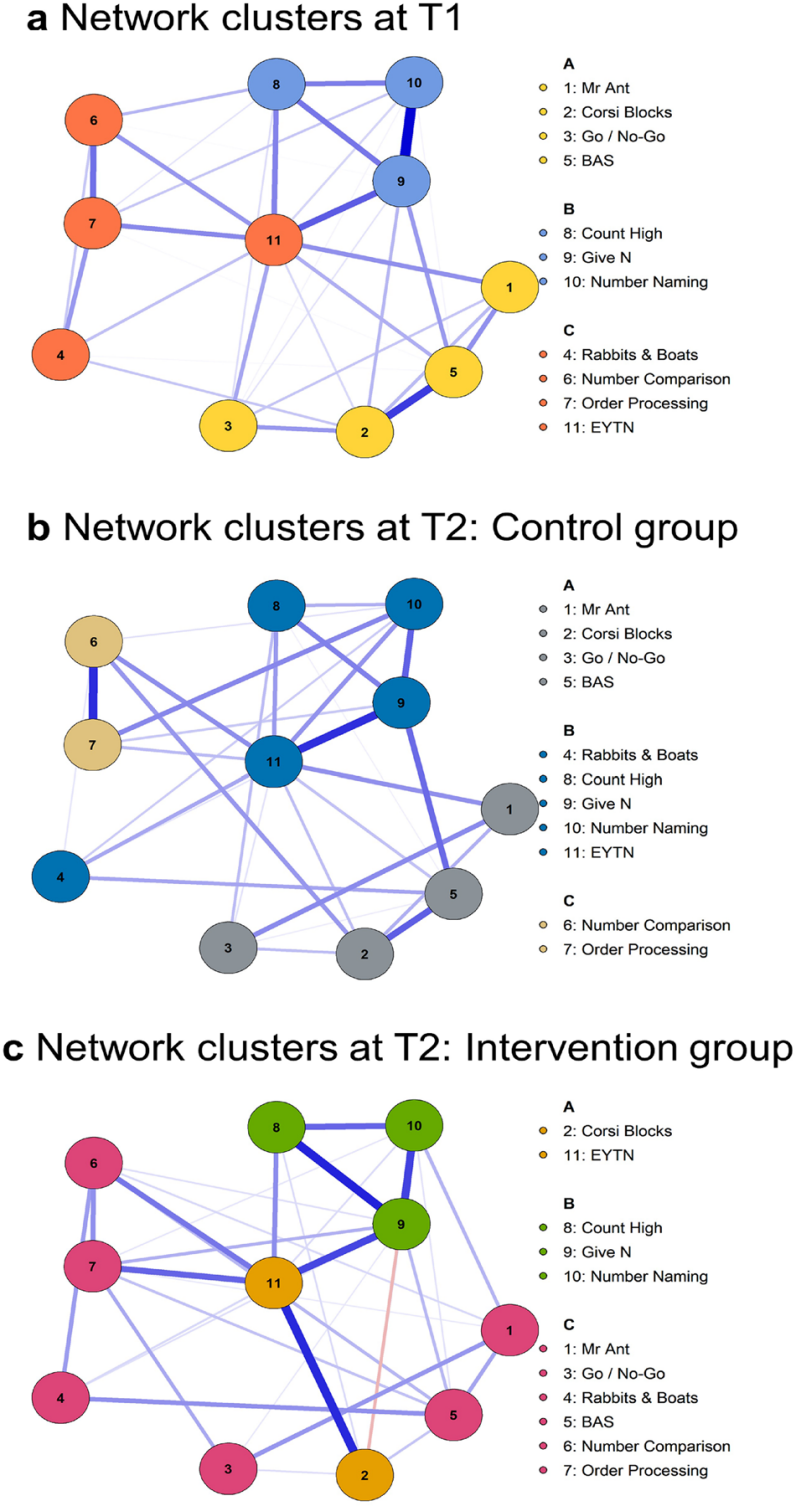
Network clusters. Network clusters (**a**) at the start of the study (T1 overall network) and at Time 2 (T2) for **b** the control group and **c** the intervention group. There were three data-driven clusters identified across the networks, with cluster membership differing across the networks.

Finally, data-driven cluster analyses identified three clusters in all three networks (Fig. 6), but the structure of clusters (i.e., the nodes which comprise each cluster) was more similar for the T1 overall network (Fig. 6a) and T2 control networks (Fig. 6b), than for the T2 intervention network (Fig. 6c). In the T2 intervention network, most EF and mathematics nodes grouped together in a big cluster (Order Processing, Number Comparison, Rabbits & Boats, Go/No-Go, BAS – PC and Mr Ant), and EYTN and Corsi Blocks formed a central cluster. Additional findings on bridge nodes and cluster differences, consistent with greater integration in the T2 intervention network, are detailed in the Appendix.

## Discussion

The present study aimed to evaluate the efficacy of an integrated EF and mathematics intervention in improving early numeracy outcomes for children. Previous research points to concurrent, longitudinal, and transactional dynamics between early numeracy and EF^6,9,11,13–15,38^, but interventions that have focused on executive functions in isolation have tended not to result in improvements in correlated functions^17,18^. Recent reviews have hypothesised that interventions integrating executive challenge within the targeted domain(s) – in this case, mathematical content – have the potential to improve early numeracy most effectively^19,20^. These proposals also connect with broader frameworks of neurocognitive development, such as interactive specialisation^27^ and mutualistic^28^ or transactional views^6,13^. Yet empirical evidence on the efficacy of integrated EF interventions, for young children in particular, has been more limited and mixed. Moreover, the relationships between specific numerical and specific EF preschool skills both prior to and following interventions have been under-investigated thus far, as most studies focus on multi-componential indices of mathematics achievement or EF factors.

We hypothesised that an integrated EF and mathematics intervention would result in improvements in mathematics. In the current study, an integrated intervention resulted in a greater differential change in an overall early numeracy measure for children in the intervention arm compared to those in the control group. Our primary hypothesis had focused on overall mathematics achievement because this has been the target of previous educational interventions and studies of transfer post-EF training, but children in the intervention group also improved more than children in the control group on EF measures, particularly for working memory indices and in the context of economic disadvantage. Our efficacy findings therefore support our hypothesis, even when modelling baseline practice-as-usual differences across settings in the level of support offered by educators to children in their care. The beneficial effects of integrating early mathematics and EF on mathematics add to a growing body of evidence in favour of integrated interventions^39^. Moreover, our study addressed calls to gather more evidence on the integration of domain-specific and domain-general co-developing skills, in order to understand the successes and failures of interventions^19,20,40^. Although we did not collect neural or long-term longitudinal data, our findings are also consistent with broader theoretical frameworks of neurocognitive development and longitudinal data that emphasise the integration of co-developing skills over time, both generally^27,28^ and specifically in the context of EF and mathematics^6,13^.

Complementing efficacy analyses that focus on variables in isolation, our network-based approach showed that there was a high degree of interconnectedness between EF skills themselves, and between EF and mathematics skills. The high degree of interconnectedness is consistent with previous data on EF^35^ and EF and mathematics^10^ in the preschool years. In addition, the interconnectedness was higher in the post-intervention network. The EF-mathematics post-intervention network for children in the intervention group differentiated from the pre-intervention network to a greater degree in terms of overall similarity, centrality indices, bridge nodes and data-driven clusters of nodes compared to the Time 2 control group’s network. These findings point to additional benefits that would not be expected from simply addressing either EF or mathematics on their own. The efficacy-based findings and network analyses provided two complementary approaches that, together, indicate that the integrated EF and mathematics intervention did not simply improve outcome variables in isolation, but also changed their interconnectedness. This might be because children practised EF and mathematics skills together to a greater degree than in practice-as-usual.

One of the key benefits of graph-theory-based approaches is that they model intercorrelations between multiple variables, rather than treating them in isolation^32^. Network analyses offer a strong complementary alternative to data reduction approaches such as exploratory or confirmatory factor analyses that have come under criticism recently^41^. This is because networks do not only model shared variance, but they also represent correlations between nodes once all others have been modelled^42^. Our findings are consistent with the increases in connectedness reported for another study investigating inter-relations between distinct EF nodes after an EF-focused intervention in older children and adolescents, with the network for children in the intervention group demonstrating network indices that were more similar to the baseline adolescent network than children in the control group^34^. The current post-intervention intervention network also differentiated from the control network at Time 2 in terms of lower overall similarity to the Time 1 overall network, it displayed greater centrality (connectedness), stronger bridge nodes and different data-driven clusters of nodes, supporting the suggestion that an intervention bringing EF and mathematics together fosters the co-development of these skills.

Understanding not only whether integrated interventions work for all children, but also whether children from different socio-economic backgrounds benefit differently from them, is very important. In the current study, children growing up in conditions of economic disadvantage scored lower on most of our numeracy and EF variables, but, when in the intervention group, they improved more than children who were also at an economic disadvantage, but in the control group. These greater benefits extended to overall numeracy, spatial skills, visual short-term memory skills and a latent EF variable. The lower performance on EF and mathematics tasks in children living in poorer socio-economic circumstances is consistent with prior research^43–45^. Risks for lower EF and mathematics performance are likely to co-occur with economic disadvantage^12,46^. However, strong EF can act as a protective buffer and predictor of good mathematics performance at school entry^15,38^. Crucially, inequalities in EF and mathematics are likely to depend on a complex host of factors, some of which may be very hard to modify (e.g., systemic barriers to access to resources, pervasive environmental stressors, etc.)^47^. However, other factors are likely to be modifiable through changes in policies and educational opportunities in early years settings (such as opportunities to practice mathematical activities^44^, support for parents^48^, high-quality early years support^49^). In this study, exposure to an integrated EF and mathematics intervention benefited the sample of children who were the most economically disadvantaged, supporting the view that greater opportunities for exposure and practice can improve both EF and mathematics in the context of economic disadvantage. These findings are also consistent with the greater success of curriculum-based interventions in improving EF and/or mathematics for children who are experiencing more economic disadvantage than for children experiencing less severe disadvantage^31,50^. We believe that curriculum-based approaches are promising for levelling the playing field early on before attainment gaps set in and widen. The approach is advantageous as it does not involve changing parenting behaviours, particularly for parents who may already be under-resourced with limited time.

Together with these positive outcomes, there are limitations and much-needed future research before we have a good understanding of integrated interventions such as The ONE programme. As a first limitation, here we contrasted the intervention regime with a practice-as-usual control group, rather than a control group engaged in a different intervention regime. We did this explicitly because ethically we felt it was most appropriate to first demonstrate the feasibility and acceptability of a newly developed intervention programme, as well as its efficacy, before contrasting it with another regime. The need for an active control group was reduced by the fact that our activities were delivered by the classroom educators, rather than a novel set of adults (e.g., researchers) who might make children’s experience very different to practice-as-usual. In addition, the activities did not involve the use of unusual manipulatives and media. This is important because it reduces the possibility that any improvement could depend on increased attention to a novel set of objects or new researchers interacting with children in each classroom. Instead, educators integrated activities into their everyday practice. In addition, we reasoned that conceiving “practice-as-usual” in educational intervention studies as “non-intervention” may in and of itself be misguided. The pre-existing educational environment on which an intervention is overlaid offers active elements that must be measured, rather than ignored. This was indeed why we characterised the educational differences across all settings, using an adaptation of standardised observational measures of the educational environment and pedagogy used in adult/child interactions (the Sustained and Shared Thinking and Emotional Wellbeing Scale, SSTEW^51^). We then modelled these differences analytically, to study whether The ONE added to variation in educational contexts.

However, future studies could compare integrated interventions such as The ONE programme directly with isolated EF interventions, to better understand whether improvements in mathematics or EF are due to the mathematical or EF elements of the intervention, or due to their integration. The additional contrast with an active, but not integrated, EF comparison regime, would further isolate the mechanisms underpinning whether and how integrated interventions are more effective. For example, a comparison group working on EF activities without mathematical content (e.g., PRSIST^37^, with a focus on EF and not integrated EF and mathematics) might show changes in EF nodes, but more limited or no changes in the edges connecting EF and mathematics nodes. A further alternative would be to contrast different integrated regimes (e.g., EF and mathematics, as in The ONE programme, and EF and another co-developing skill, such as oral language) in terms of their general and specific benefits to EF and the skills with which EF is integrated. At present, our findings point to preschool EF and mathematics as sets of processes being in a state of dynamic interplay as shown by all our networks. On the whole, while we believe that independent effects of mathematics training and EF training may be empirically tractable and statistically measurable, interactions and dynamics best reflect both longitudinal data from other studies and our intervention effects.

A second limitation is that our study was not designed to explicitly pit against each other different latent factor accounts of EFs (e.g., a unitary vs differentiated model), as measuring different later factors would have required at least two EF observed indices per component EF skill^3,4^. Our protocol aimed to provide breadth in both EF and mathematics, and it was therefore simply unfeasible to test our very young children with many more EF tasks pre- and post-intervention, in the time available. However, by virtue of network analysis, we do report additional and novel relationships between observed (although not latent) EFs with each other. Within the limited context of our four observed variables, EF indices were highly correlated with each other, as previously reported for pre-schoolers^3,4^, suggesting that either measuring separable EFs in this age group is very hard with the current measures available, or simply that EFs cluster together much more closely at this stage. In addition, Corsi Blocks played a central role as a node within the EF network, a very interesting finding because other network analyses of EF later in life have pointed to cognitive flexibility having increasing centrality in older childhood and adolescence^35^. Furthermore, distinct EF components clustered differently with component mathematics skills before the intervention compared to later, suggesting both unity and diversity in the relationships between EF and mathematics. In turn, this diversity is consistent with recent longitudinal data in the primary school years^6^. Network analyses have recently been used to study age-related differences in the structure of EFs from childhood and across the lifespan^35,36^. To our knowledge, these methods have not been employed to investigate EF and mathematics networks at any age. Future work in pre-schoolers will need to measure larger samples and an even greater number of age-appropriate EF and mathematical tasks than we did, to study relationships between both observed measures of EF and mathematics, and in latent factor structures of different complexity.

A third needed future direction is to replicate the intervention benefits for children growing up in conditions of economic disadvantage by broadening how we approach disadvantage. By using EYPP eligibility as an index of disadvantage, the disadvantage here was simply operationalised as low income. A broader operationalisation, going beyond low income only, is needed^52^. It would be helpful to extend this to look at parental education, family resources, cultural practices related to learning, and the quality of early years setting. Furthermore, in the current study, benefits for children at economic disadvantage varied across indices of mathematics and EF: in the context of disadvantage, children exposed to The ONE programme benefited more than children in the control group on overall early numeracy, spatial processing and visual short-term memory indices, but not on other measures. Potential explanations start with measurement considerations: perhaps children at economic disadvantage had more “room to grow” on these measures. However, explanations also extend to greater “integration practice”: perhaps integrating space and shape games with EF may have occurred more frequently than in practice-as-usual, in particular for disadvantaged children in the intervention. The differential stronger benefits for some skills compared to others require further formal investigation.

Finally, a further required step is to test whether the current benefits of The ONE programme are replicated in a larger sample of diverse children and settings. This is because the current sample of disadvantaged children was relatively small, although it exceeded the national United Kingdom average of EYPP eligibility. Furthermore, here we could only control for, but not model explicitly, the impact of diversity across settings. A replication of the programme with a larger number and more varied types of preschool settings is important to examine the interplay between children’s characteristics, preschools’ characteristics, and intervention success. A future large-scale trial is necessary to test these multiple factors and their interplay with sufficient statistical power. This will allow for a greater understanding of whether and how the intervention is most effective when it has gone to scale.

In conclusion, executive functions are known to correlate strongly and robustly with co-developing functions such as early mathematical skills, but interventions that have focused on training EF in isolation have thus far failed to show reliable improvements in early mathematics. Interventions that integrate EF with co-developing functions hold more promise, but greater evidence about their efficacy, particularly for children growing up at a disadvantage, and a better understanding of their mechanisms, are required. Here, network analyses pointed to greater changes in the EF-mathematics interplay associated with the intervention than with the simple passage of time. In combination, these findings point to the need to carefully consider and leverage the interplay between EF and co-developing cognitive domains, rather than intervening on these cognitive functions in isolation.

## Methods

### Ethics approval statement

This cluster randomised controlled trial (RCT) received research ethics approval from the Central University Research Ethics Committees of the University of Oxford (R68839/RE008: Fostering Resilience by injecting executive challenge into early maths). Early Years education settings opted into the study after receiving information about all its elements. Parents and guardians decided whether to opt out of the study by communicating this to settings, preserving their anonymity. Although informed consent to take part in studies is a frequent mode of consent, the research ethics committee waived this requirement exceptionally in this case and permitted the opt-out model of participation because it is more likely to represent families and children from socio-economically disadvantaged backgrounds in longitudinal designs^53^.

### Participants: children and settings

The study sample consisted of 193 children (Mage at baseline = 47.2 months, range = 41–54; 111 females; reported ethnicity: 69% White, 16.1% Asian, 10.3% Multiple Ethnic Groups, 2.3% Black, 2.3% Other). Child demographics by intervention and control group are reported in Table 3. Economic disadvantage was identified by using eligibility for Early Years Pupil Premium (EYPP). Eligibility for this programme in England includes a family annual income below GBP 16,190 and/or meeting other high-risk criteria (e.g., asylum seeker status). EYPP eligibility is, therefore, an index of economic disadvantage, although it may underestimate disadvantage because parents who are eligible do not all apply (for reasons associated with stigma, social desirability, and/or administrative barriers in the application process). EYPP eligibility was assessed based on reporting by the child’s nursery school (*N* = 147) and parent-reported income (*N* = 77). Of the 161 children (83.4% of the sample) for which these data were available, 24.8% (*N* = 40) were deemed eligible for EYPP (higher than the 14% national UK average for 2022). Of note, when the study was conducted, all 3- to 4-year-olds in England were eligible for at least 15 h of free preschool, whether they attended a private setting or not, making preschool an appropriate environment to target disadvantaged, because preschool was accessible to all. The control group and the intervention group were well-matched in terms of age in months, sex, EYPP eligibility and school readiness (see Table 3).

**Table 3:** Summary of demographic information for control and intervention children

Measure	Control	Intervention	Difference
Number of participants (*N*)	90	103	
Age pre-intervention (months, SD)	47.2 (0.36)	47.3 (0.37)	n.s. (*p* = 841)
Sex (% female)	56.7	58.3	n.s (*p* = 824)
EYPP eligibility (% eligible)	21	28	n.s. (*p* = 406)
Special educational needs (SEND) (%)	6.7	4.9	n.s. (*p* = 497)
English spoken at home (%)	80	68	n.s. (*p* = 155)
Average BESSI score	1.16	1.19	n.s. (*p* = 329)

For children for whom information was not returned, the data were treated as unknown. Ethnicity was reported by (voluntary) completion of a parent questionnaire. 87 parents (45%) returned this information. The Brief Early Skills and Support Index (BESSI, Hughes et al.,^63^) is a teacher-reported measure of school readiness, with higher scores representing lower school readiness. Items cover: (1) behavioural adjustment, (2) language and cognition, (3) daily living skills, and (4) family support. Each item is given a score of 1 (strongly agree or agree) or 2 (strongly disagree or disagree), with a higher score representing more problem behaviours.

**Fig. 7:**
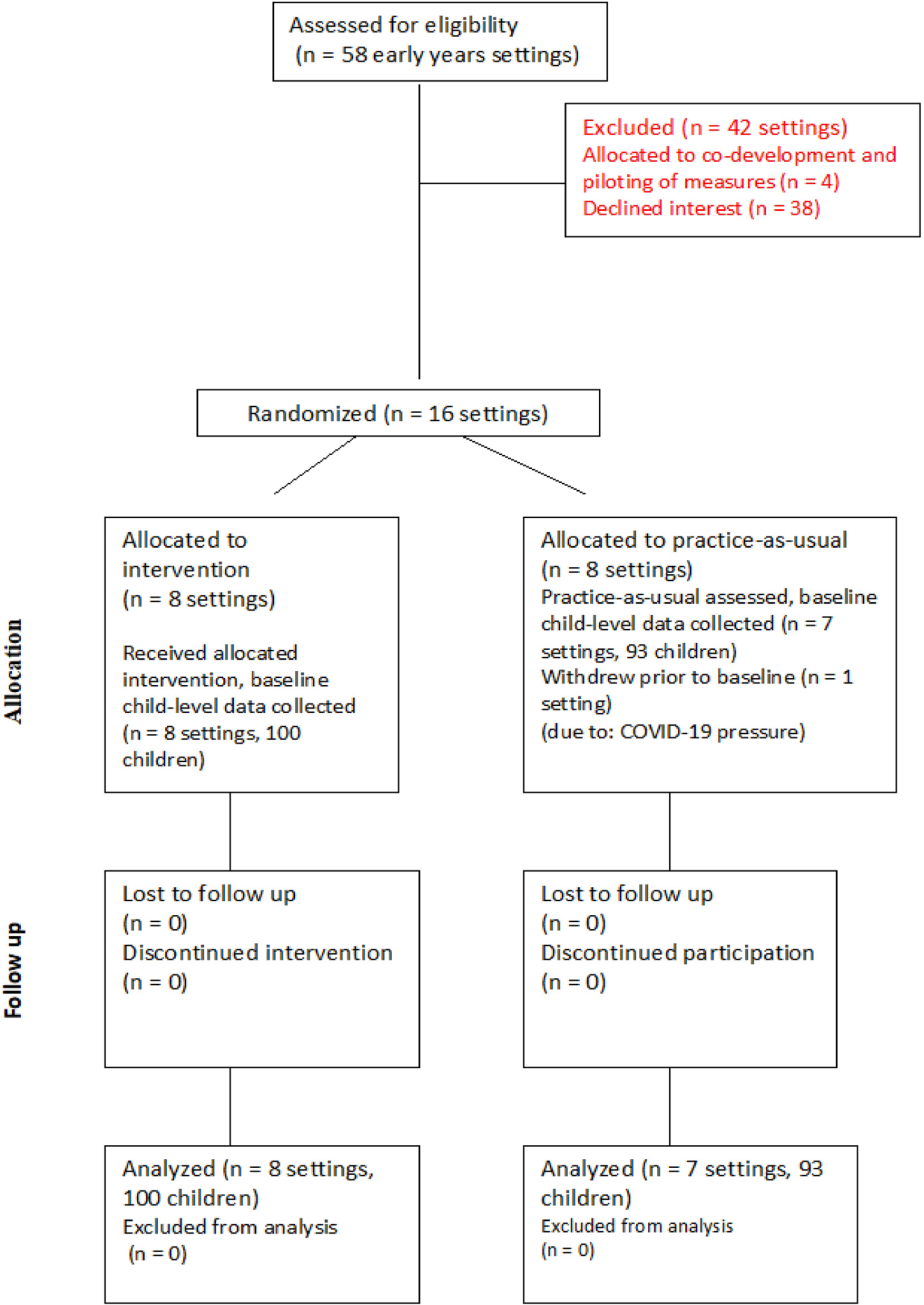
CONSORT diagram. CONSORT diagram describing the flow of the study, from recruitment to endline assessment.

Fifty-eight settings were approached to take part in this research on the basis of geographical spread and feasibility of travel from Oxford, of which 20 (34.5%) consented to take part (see CONSORT diagram, Fig. 7). Four of those services took part in an initial co-development phase of the research, with the other 16 participating in this RCT evaluation of The ONE Programme reported here. Settings were randomised to either the control group or the intervention group by a research team member who had not interacted with any of the settings, stratifying on the basis of setting size (large/small), setting type (private or not) and UK-based neighbourhood disadvantage metrics (the Indices of Multiple Deprivation (IMD) deciles and Income Deprivation Affecting Children Index (IDACI) based on the postcode of the preschool). The process allocated 8 settings to the intervention and 7 to the control group (one control setting withdrew before the pre-intervention baseline due to ongoing COVID-19 pressures), well-matched on stratification variables (see Table 4).

**Table 4:** Summary of characteristics for control and intervention settings

Measure	Control settings	Intervention settings	Difference
Number of settings	7	8	
IMD (SD)	5.5 (2.62)	5.5 (2.14)	n.s. (*p* = 1.0)
IDACI (SD)	5.0 (2.39)	5.0 (2.82)	n.s. (*p* = 1.0)
Average number of children per setting (SD)	30.7 (25)	30.9 (21)	n.s. (*p* = 725)
Setting type (% private)	62.5	42.9	n.s. (*p* = 317)

Characteristics of settings volunteering into the study.

*IMD* Index of Multiple Deprivations, Indices of Multiple Deprivation decile, *IDACI* Income Deprivation Affecting Children Index decile, with scores ranging from 1 to 10, with lower scores indexing greater deprivation.

In addition, prior to randomisation and to pre-intervention child assessments, settings were observed via a half-day observation of the interactional quality of the Early Years environment and interactions, using the Sustained and Shared Thinking and Emotional Wellbeing Scale (SSTEW^51^). The SSTEW scale was developed to assess the quality of interactions between adults and children in early years childhood education settings, and its overall score predicted early numeracy indices in a large sample of Australian pre-schoolers^51^. We supplemented SSTEW with bespoke mathematics observation items, capturing interactional quality in the context of counting and cardinality, shape and spatial awareness, patterning and ordering, and numerical knowledge. This observation schedule yielded a score per setting that allowed us to evaluate setting comparability in the adult support that was already provided to children in each setting. In addition, it allowed us to model the effects of our integrated intervention on children while controlling for how children were nested in settings that varied in terms of baseline interactional quality (see Analysis Plan). Settings in the control and intervention groups were well-matched in terms of the quality of Early Years interactions (*M*_intervention_ = 4.27, SD = 1.48; *M*_control_ = 4.01, SD = 1.85).

### Procedure: intervention, control group, pre- and post-intervention assessments

The intervention protocol was co-developed with early years practitioners and consisted of: four weekly 30-min face-to-face interactive workshop-style PD sessions with Early Years Practitioners followed by eight weeks remaining weeks. The four sessions supported practitioners’ explicit understanding of how early mathematics and EF co-develop, introduced 25 Mathematics + EF activities and explained how EF can be embedded into a range of routine early mathematics learning activities. All activity cards described their mathematical content and executive demands explicitly. The activities ranged from EF-enhanced modifications of common early childhood games (e.g., “What’s the Time Mr Wolf?”, with embedded executive demands – e.g., “We do not walk if Mr Wolf says… ‘it’s 2 o’clock’”), to more novel activities introducing challenge in EF and mathematics through play (e.g., “Number Robot”, a handmade cardboard function machine requiring cognitive flexibility to apply mathematical functions^54^). All activities started with mathematical content and EF challenge at a base level. Instructions and training were provided to scale complexity as the activities became familiar to children.

Activities were designed to use low-cost and readily available materials. In consultation with pilot settings and early years specialists, the activities were explicitly designed to be chosen flexibly each week by teachers, rather than in a fixed order, to suit each setting’s context, given the diversity of setting types (e.g., presence or absence of outdoor space, preference for small or large group activities), thereby maximising acceptability and feasibility. Preschool staff were asked to implement a minimum of three of these activities per week with 3- to 4-year-old children at their setting, for the 12-week duration of the programme. The intervention was carried out at the whole-class level and was not targeted towards specific groups of children.

Despite flexibility and choice, there were core demands made of all educators, and these core demands reflected the theory of change of the intervention that was explicitly explained to classroom educators. First, the three activities undertaken within a week should be chosen to target breadth in mathematical content, by choosing one activity in each of the three key areas of mathematics represented in the activity pack (numbers and counting, patterns and ordering, space and shapes). Practitioners were asked to play the activities in their basic form in weeks 1–8 of the programme, but in Week 8 they were reminded to increment the executive challenge of chosen activities as children became increasingly familiar with them. In addition to the recording of activities on a poster provided to log adherence, one representative per setting was contacted in the 8th and 12th weeks to enable practitioners to reflect on how the programme was going, to enable a member of the delivery team to provide support, and, in Week 12, to conduct an interview (establishing acceptability and barriers of the programme) and an observation (to check fidelity of delivery).

We compared the group of children nested in settings receiving the intervention to a practice-as-usual control group of children who received standard early years education following the Early Years Foundation Stage curriculum in the United Kingdom. We were specifically interested in whether the programme improved children’s mathematical skills above and beyond teaching in mathematics that is already embedded in the curriculum. As the intervention took place in early years settings, children and educators in the practice-as-usual settings were not passive: children in this group received instruction and teaching by their educators, following a standard practice that aims to foster socio-emotional self-regulation and mathematical skills as set out in the curriculum. We aimed to capture these practices across all settings via structured observations while contrasting explicit EF and mathematics integration to practice-as-usual levels of integration. Our trial design was in line with education trials, guided by policy-makers and practitioners, who want to know whether a programme works above and beyond usual practice.

All children were tested individually across two 30-min sessions, counterbalanced across children, on two separate days, both before and after the intervention period. Random assignment to either the intervention or practice-as-usual arm occurred after completion of baseline data collection. Post-intervention child-level assessments were carried out by researchers who were blind to trial arm allocation, on average 5 months after the pre-intervention assessments.

#### Mathematics. General numeracy - early years toolbox – numeracy^55^

The early years toolbox numeracy (EYTN) task is a tablet-based measure of general numeracy skills. Interspersed items on the task pertain to various mathematical domains: number sense, cardinality and counting, numerical operations, spatial and measurement constructs and patterning. The total accuracy score was used for analysis, with one point scored for each correct item.

#### Specific mathematical skills. Count High^9^

To assess children’s counting skills, children were instructed to count as high as they could and the highest number reached without having made any mistakes was recorded, stopping at 100 as maximum. ***Give N (adapted from ref.*** ^56^***)****.* A version of the Give-N task was used as a measure of cardinality, following the adapted procedure outlined by ref. ^56^. Children were asked to place a given number of plastic fruit on a plate for 3 blocks of 5 trials, using numbers 3, 4, 6, 11 and 15. The final score was the number of correct trials out of a possible 15. ***Number Comparison (adapted from ref.*** ^57^***)***. This task is designed to measure children’s digit comparison abilities. Two number digits (1–9) were presented side by side on the screen of a tablet and the child was asked to tap the larger of the two numbers. The final score was calculated as a proportion of numbers correct out of all items answered within 1 min. ***Number naming***^57^. As a measure of symbolic number knowledge, children were presented with each digit from 1 to 9 twice on a screen in a random order, resulting in 18 total digits. The researcher pointed at each digit in turn, asking the child, “What number is this?”. The score used was the number of correct items out of a possible 18. ***Order Processing***^56^. Children were presented with a set of three number cards, each containing one Arabic numeral (1–9), which they were asked to place in order from smallest to biggest. Following 4 practice trials, there were 12 main trials. The task ended after six cumulative mistakes. A total score out of 12 was calculated for analysis. ***British Ability Scale - Pattern Construction****.* The pattern construction scale from the third edition of the British Ability Scale (BAS3), was used as a measure of spatial ability. This scale requires children to copy spatial patterns using wooden blocks, foam squares and plastic cubes with different patterned and coloured sides. A standardised t-score based on the child’s age in months was used for analysis.

#### Executive function. Corsi blocks task (following ref. ^46^)

This is a measure of children’s visuospatial short-term memory. Nine wooden blocks were attached to a white piece of cardboard in a random array. The researcher tapped blocks in a pre-set random order and the child was instructed to tap the same blocks. For each span level (e.g., 2 block-sequences), the child completed 3 trials. If 2 or more trials were correct, the child progressed onto the next span level (up to 6 block-sequences). The variable used for analysis was the overall number of correct trials, regardless of sequential order. ***Mr Ant***^58^ is a visuospatial memory task presented on a tablet in which the child is asked to remember the location of colourful ‘stickers’ placed on different body parts of a cartoon ant. In each trial, the stickers are presented one after the other. A blank ant then reappears and the child is asked to indicate where the stickers had previously been, by tapping those locations. There are three trials in each block, with the child progressing to the next block if they are correct on at least one trial, regardless of sequential order. A score was calculated as one point for each consecutive level, beginning from the first, with 2 or 3 items correct; then, from the first level with only 1 item correct, 0.33 points for each correct item. ***Rabbits & Boats***^58^ is a tablet-based shifting task, based on a traditional card sort task. Across three blocks, the child must sort cards first according to colour (red/blue), then to shape (rabbit/boat), and finally switching the rule depending on whether or not there is a black border. Each block contains 6 trials and the child must get at least 5 trials correct on blocks 1 and 2 in order to progress to block 3. A switch accuracy score, calculated as the sum of correct responses in blocks 2 and 3, was used for analysis. ***Fish-Shark Go/No-Go***^58^ is a tablet-based task of inhibitory control. Fish and sharks move across the screen, one by one in pseudo-random order, and the child is instructed to tap the fish (go trials) and not tap the sharks (no-go trials). There were 3 blocks of 25 trials, each consisting of 20 go trials and 5 no-go trials. Proportional go and no-go accuracy scores were multiplied to create an overall impulse control score, which was used for analysis. Information on reliability for these measures is detailed in the Supplementary Online Materials, for brevity.

In addition, data reduction (via exploratory factor analysis) was employed to calculate an **overall index of EF** for this sample (in line with the existing literature in this age group^3,9^). A single factor with an Eigenvalue greater than 1 was identified, accounting for 47% of the variance in EF scores, and ***EF latent factor scores*** were produced. The goal of this factor analysis was to provide an index for EF that would be comparable to our variable for overall numeracy, EYTN. Of note, we had not set out to explicitly test against each other the fit of single latent factor accounts of EF versus multiple latent EF factors, as this has been done previously by other researchers by using a much larger complement of EF tasks (e.g., at least two observed indices per construct^3,4^). Of note, although most commonly employed in the literature to date, computing latent factors is not the only approach that one could use to calculate a multi-factorial EF index, with the calculation of EF composite scores holding complementary merits^41^. This was indeed such an interesting question that we explored calculating a composite score (as the averaged standardised performance scores for our four EF tasks). This composite score correlated highly with the latent factor score (rho = 0.965, *p* < 0.001), and therefore for brevity, we report findings using the latent factor score.

### Data analysis plan: transparency and openness section

We pre-registered the trial design and measures on Open Science Framework prospectively before data were collected [https://osf.io/8y5u6/]. As recommended by the APA Journal Article Reporting Standards (JARS) for quantitative, qualitative, and mixed methods research, we report how we determined our sample size, all data exclusions (no data exclusions were employed), all manipulations (no data transformations were employed), and all measures in the study. Anonymized data and analysis code are available at [https://osf.io/8y5u6/]. Our planned child-level efficacy outcomes variables were early mathematics and EFs measures, as reported at [https://osf.io/8y5u6/]. An intention-to-treat analytical approach (with all children in settings that had been randomised to the intervention included in the intervention arm) was employed, consistent with other educational trials. The efficacy analysis was carried out using IBM SPSS v 29.0. The network analyses were exploratory and were conducted in R statistical software (version 4.2.2) using packages *qgraph* (version 1.9.3^59^), *bootnet* (version 1.5^60^) and *networktools* (version 1.5.0).

#### Pre-registered intervention efficacy analyses

The target sample size (*N* = 240 children) was determined a priori using G*Power 3.1^61^ to afford power greater than 80 to detect a small (*f* ^2^ = 0.10, as expected for educational intervention) interaction effect for intervention arm (integrated, BAU) and time point (pre-intervention, post-intervention), with alpha = 0.05, repeated measure correlation of 8, with up to 20% attrition. Due to ongoing COVID-19 impact (e.g., nursery staff turnover, lower time availability for settings), one setting withdrew from the study before pre-intervention assessments and parents of one child withdrew data from the study. The final *N* was *N* = 193. No data were excluded. **Deviations from pre-registration**. We had planned to use two-way mixed ANCOVAs, but missing data (average univariate missingness = 5.8%; maximum univariate missingness = 17.6%) and distributional violations required approaches that deviated from the pre-registered analyses. Multi-Level Linear Modelling (MLM) with restricted maximum likelihood estimation (REML) was employed to model main effects over and above Time 1 individual differences, because this is robust to moderate to small proportion of missing data and to distributional violations^62^. As described by Eq. (1) below for one of our outcome variables (EYTN), MLMs modelled the effects of Time (Time 1, Time 2), Intervention group (Control, Intervention), and Early Years Pupil Premium (EYPP) eligibility (EYPP; Yes, No, Unknown). Time and participant data were modelled as repeated effects. Setting-level differences in baseline scores for interactional quality (SSTEW, Siraj et al., 2015) were modelled as random effects. Nesting of children-level data within settings was employed to model setting-level variables (baseline differences in interactional quality as above, SSTEW) and child-level variables (EYPP eligibility). We computed the effect size using Hedge’s *g*.

1 $${{EYTN}}_{{ij}}=\alpha +{\beta }_{1}{{Treat}}_{i}+{\beta }_{2}{{EYPP}}_{i}+{\beta }_{3}{{Time}}_{i}+{\beta }_{4}{{Treat}}_{i}* {{EYPP}}_{i}+{\beta }_{5}{{Treat}}_{i}* {{Time}}_{i}+{\beta }_{6}{{Time}}_{i}* {{EYPP}}_{i}+{\beta }_{7}{{Treat}}_{i}* {{EYPP}}_{i}* {{Time}}_{i}+{p}_{i}+{{sstew}}_{j}+{{\rm{\varepsilon }}}_{{ij}}$$

Where *i* are children and j are preschool settings; EYTN_ij_ is the EYTN Score; $\alpha$ is the overall intercept; β_1_Treat_i_ is the fixed effect of the treatment indicator for child *i*; ${\beta }_{2}{{EYPP}}_{i}$ is the fixed effect of the EYPP eligibility for child *I*; β_3_Time_i_ is the fixed effect of Time for student *i*; β_4_Treat_i_ * EYPP_i_ is the interaction effect between treatment and EYPP eligibility; β_5_Treat_i_ * Time_i_ is the interaction effect between treatment and Time; β_6_Time_i_ * EYPP_i_ is the interaction effect between EYPP eligibility and Time; and ${\beta }_{7}{Trea}{t}_{i}* {{EYPP}}_{i}* {{Time}}_{i}$ is the interaction effect of treatment, EYPP eligibility and time. *p*_i_ is the random effect of child *i*, sstew_j_ is the random effect of SSTEW score per preschool setting *j*, and Ɛ_ij_ is the residual error term. All other models replaced EYTN for the other dependent measures but tested the same main effects and interaction effects.

#### Exploratory network analyses

To explore the structure of the relationships between all EF and mathematics variables at once, rather than focusing on bivariate correlations or univariate changes from pre- to post-intervention, we implemented Gaussian graphical network models based on a regularised partial correlation network using Spearman correlations^42^. The EF and mathematics tasks were represented as nodes in each network, while the partial correlations between the tasks represented the network edges (i.e., connections between nodes). To test whether this integrated intervention led to greater changes in the network structure than practice-as-usual, we tested overall network change by calculating the correlation coefficients between all edges of the network (i.e., the connections between the nodes) pre- and post-intervention, in the intervention and the control group. To further characterise the estimated networks, we tested the relative importance of each node in the network by calculating *centrality indices*: strength, expected influence, closeness, and betweenness all characterise the connectedness of nodes in a network. The Strength index refers to the absolute sum of all edges (i.e., correlations) to a particular node (e.g., all paths to a mathematics node). In contrast, Expected Influence takes into account whether an edge (a correlation) has a particular sign (positive or negative). Betweenness refers to how often a node is on the shortest path between other nodes, and Closeness refers to the mean distance from a node to all other nodes in the network. Additional node and edge stability are reported in Supplementary Figs. 4 and 5. In addition to interrogating the importance of individual nodes in the network, we tested whether there are any prominent *bridge* nodes between EF and mathematics nodes, i.e., nodes in one group that are most strongly connected to all nodes from the other group. The detection of bridge nodes enabled us to determine the strongest links between domains, i.e., which EF node was most strongly connected to mathematics nodes, and vice versa. Finally, to determine whether there were clusters of nodes in the network and whether the cluster structure changed with the intervention, we ran a cluster analysis. In graph-based approaches, the presence of clusters shows that some nodes are more strongly related than others and it is determined via a data-driven approach.

## Supplementary information

Supplementary Materials

## Data Availability

The data necessary to reproduce the analyses presented here are available on Open Science Framework [https://osf.io/8y5u6/]. A full description of the baseline and endline assessment materials is also available on Open Science Framework [https://osf.io/8y5u6/]. The intervention efficacy analyses were pre-registered on the Open Science Framework before data collection began [https://osf.io/8y5u6/].

## Code availability

The analytic code necessary to reproduce the analyses presented here is available on Open Science Framework [https://osf.io/8y5u6/].
