# Supplementary material for: Enhancing children’s numeracy and executive functions via their explicit integration
Source: NPJ Sci Learn. 2025 Feb 18;10:8. doi: 10.1038/s41539-025-00302-9 (PMC11836118; doi:10.1038/s41539-025-00302-9)
Supplement: Supplementary file 1 — Supplementary Materials [file 41539_2025_302_MOESM1_ESM.pdf]

Supplementary Online Materials

Additional details on The ONE intervention

The intervention was co-developed with early years practitioners and consisted of: four weekly 30-minute face-to-face interactive workshop-style professional development sessions with Early Years Practitioners followed by eight weeks of the programme. These four sessions supported practitioners' explicit understanding of how early mathematics and EF co-develop, introduced 25 Mathematics + EF activities, and explained how EF can be embedded into a range of routine early mathematics learning activities. All activity cards described their mathematical content and executive demands explicitly. The activities ranged from EF-enhanced modifications of common early childhood games (e.g., "What's the Time Mr Wolf?", with embedded executive demands – e.g., "We do not walk if Mr Wolf says... 'it's 2 o'clock'"), to more novel activities introducing challenge in EF and mathematics through play (e.g., "Number Robot", a handmade cardboard function machine requiring cognitive flexibility to apply mathematical functions, Moss et al., 2016). All activities started with mathematical content and EF challenge at a base level. Instructions and training were provided to scale complexity as the activities became familiar.

Activities were designed to use low-cost and readily available materials. In consultation with pilot settings and early years specialists, the activities were explicitly designed to be chosen flexibly each week by teachers, rather than in a fixed order, to suit each setting's context, given the diversity of setting types (e.g., presence or absence of outdoor space, preference for small or large group activities), thereby maximising acceptability and feasibility. Preschool staff were asked to implement a minimum of three of these activities per week with 3- to 4-year-old children at their setting, for the 12-week duration of the programme. The intervention was carried out at the whole-class level and was not targeted towards specific groups of children.

Despite flexibility and choice, there were core demands made of educators, and these core demands reflected the theory of change of the intervention that were explicitly explained to classroom educators. First, that the three activities undertaken within a week should be chosen to target breadth in mathematical content, by choosing one activity in each of the three key areas of mathematics represented in the activity pack (numbers and counting, patterns and ordering, space and shapes). Following the model developed by PRSIST, practitioners were asked to play the activities in their basic form in weeks 1 – 8 of the programme, but in Week 8 they were reminded to increment the executive challenge of chosen activities as children became increasingly familiar with them. In addition to the recording of activities on the poster we provided, one representative per setting was contacted in the 8th and 12th weeks to enable practitioners to reflect on how the programme was going, to enable a member of our team to provide support, and, in Week 12, to conduct an interview (establishing acceptability and barriers of the programme) and an observation (to check fidelity of delivery).

Additional Details on pre- and post-intervention assessments

Pre-intervention assessments took place half-way through the year preceding entry into school. Post-intervention child-level assessments were carried out by researchers who were blind to trial arm allocation, on average 5 months after the pre-intervention assessments, two or three months before entry into school.

Numeracy.

General numeracy (Early Years Toolbox - Numeracy[1]). The early years toolbox numeracy (EYTN) task is a tablet-based measure of general numeracy skills. Children responded to a series of problems presented on the iPad by either tapping the screen (e.g. "Tap the largest tree") or providing a verbal response, which the experimenter then records in the app (e.g. "How many cats are there?"). The EYTN was validated on a set of 246 Australian 3-5-year-

olds, and showed good concurrent validity with comprehensive and commercial tests of early numeracy and good test-retest reliability (r (46) = .89)[1].

Specific numerical skills. Count High[2]. To assess children's counting skills, children were instructed to count as high as they could and the highest number reached without having made any mistakes was recorded, stopping at 100 if the child was able. This task has previously been undertaken with British preschoolers[2], when it showed good sensitivity to change across a 5-month period. ***Give N (adapted from[3]).*** A version of the Give-N task[4] was used as a measure of cardinality, following the adapted procedure outlined by Cahoon et al[3]. Children were asked to place a given number of plastic fruit on a plate for 3 blocks of 5 trials, using numbers 3, 4, 6, 11 and 15. Once the child had placed the items on the plate, the researcher asked "Is that [n]?". If the child responded "Yes", the researcher proceeded to the next trial. If the child responded "No", the researcher repeated the original request. For higher numbers (6, 11 and 15), the child was asked "Can you count and make sure it's [n]?". On a similar task, Batchelor et al.[5] reported Cronbach's alpha of .76. ***Number Comparison (adapted from Nosworthy et al.[6]).*** This task is designed to measure children's digit comparison abilities. This task has previously been undertaken with British preschoolers[2]. ***Number naming[6].*** As a measure of symbolic number knowledge, again this has previously been undertaken with British preschoolers[2]. ***Order Processing[3].*** Children were presented with a set of three number cards, each containing one Arabic numeral (1-9), which they were asked to place in order from smallest to biggest. These three numbers were either sequential (1,2,3), with a gap of one (1,3,5) or with a gap of two (1,4,7). This task was previously used with a large number of British pre-schoolers[3]**.** ***British Ability Scale - Pattern Construction.*** The BAS3 was standardised on a sample of British children including 269 3- or 4-year-olds. The reported corrected Rasch split-half reliability for the pattern construction scale was .89[7].

Executive Function. Corsi Blocks Task (following[8]). This is a measure of children's visuospatial short-term memory. Nine 2x2cm wooden blocks were attached to a white A4 piece of cardboard in a random array. The researcher tapped blocks in a pre-set random order and the child was instructed to tap the same blocks. For each span level (e.g., 2 block-sequences), the child completed 3 trials. If 2 or more trials were correct, the child progressed onto the next span level (up to 6 block-sequences). Each trial was coded for whether the blocks tapped were correctly and whether the child had tapped the blocks in the correct sequential order. The variable used for analysis was the overall number of correct trials, regardless of sequential order. The test-retest reliability of this task has been shown to be very good (ICC: .90; Alloway & Passolunghi, 2011) and it has been successfully used in number of studies on preschools[8,9]. ***Mr Ant (Early years toolbox[10])*** is a visuo-spatial memory task presented on a tablet, in which the child is asked to remember the location of colourful 'stickers' placed on different body parts of a cartoon ant. In each trial, the stickers are presented for 5s, followed by a blank screen for 4s. A blank ant then reappears and the child is asked to indicate where the stickers had previously been, by tapping those locations. This task was normed on Australian preschools, and like the two remaining EF tasks, its scores have good reliability and external validity against the NIH toolbox[10]. ***Rabbits & Boats[10]*** is a tablet-based shifting task, based on a traditional card sort task. Across three blocks, the child must sort cards first according to colour (red/blue), then according to shape (rabbit/boat), and finally switching the rule depending on whether or not there is a black border. Each block contains 6 trials and the child must get at least 5 trials correct on blocks 1 and 2 in order to progress to block 3. A switch accuracy score, calculated as the sum of correct responses in blocks 2 and 3, was used for analysis. ***Fish-Shark Go/No-Go[10]*** is a tablet-based task of inhibitory control. Fish and sharks move across the screen, one by one in pseudo-random order, and the child is instructed to tap the fish (go trials) and not tap the sharks (no-go trials).

There were 3 blocks of 25 trials, each consisting of 20 go trials and 5 no-go trials. In the data cleaning process, data were removed for trials with a response time of less than 300ms, and for blocks indicating(a) non-responsiveness (go accuracy of <20% and no-go accuracy >80%) or (b) indiscriminate responsiveness (go accuracy of >80% and no-go accuracy of <20%). After these trials/blocks had been removed, proportional go and no-go accuracy scores were multiplied to create an overall impulse control score, which was used for analysis.

Additional details on Analyses Plans

Acceptability and Feasibility Analyses

Acceptability of the programme was indexed via qualitative themes emerging from end of programme interviews, conducted by reflexive thematic analysis, and via educators' rating for each activity (activities were each rated by a score out of 3). Feasibility was indexed by adherence to the programme structure, by the ability of nursery staff to deliver the required dosage of activities, and by fidelity of delivery. Adherence was recorded as the percentage of professional development sessions attended by staff at each setting. Dosage was recorded via a poster, on which, for each of the 12 intervention weeks, educators recorded which activities they completed, with the opportunity of additional qualitative feedback (e.g., activity success using a three-point smiley face scale). Fidelity of delivery was captured by a structured observation carried out at each preschool, during which an educator chose and led an intervention activity of choice out of the 25 that were part of The ONE.

Intervention Efficacy Analyses

Our planned child-level outcomes were early mathematics and EF. We had planned to use two-way mixed ANCOVAs to test the effects of intervention on mathematics and EF indices. However, after data collection, we found that a small percentage (5.78% on average) of data attrition at the univariate level would have led to much reduced statistical power for analyses

such as ANCOVA, as these result in listwise deletion of participants if they have any missing data. In addition, some data distributions violated assumptions of normality. These factors required approaches that deviated from the pre-registered analyses. Multi-Level Linear Modelling (MLM) with restricted maximum likelihood estimation (REML) was employed to test intervention efficacy because this is robust to moderate to small proportion of missing data, and to distributional violations[11]. With MLM, all the existing data were used, with Time 1 and Time 2 as a higher-level correlated factor, within which individual participants are nested. Not all datapoints are present, but the model can cope with missingness using all the remaining data, rather than imposing listwise deletion, as instead ANCOVA/ANOVA would.

Network Estimation Procedure and Parameters

As mentioned in the Methods section, we implemented network models to understand the relationship between all EF and maths variables at once, rather than looking at bivariate correlations in isolation to other relationships between the two domains. Gaussian graphical models (GGM) were implemented with a regularised partial correlation network. GGMs represent a type of Markov Random Fields suited for continuous data and are undirected, i.e., while the model estimates the strength and valence of an edge (connection between two nodes), there is no assumption about the direction of the influence between the nodes. We used Spearman correlations, as they are more robust in terms of departures in data distribution. The network edges were estimated on partial correlation matrix, hence the edge between two nodes (correlation between two variables) was estimated while controlling for all other correlations between these two nodes and other nodes in the network. We used a combined gLASSO (graphical least absolute shrinkage and selection operator) regularisation and EBIC (extended Bayesian information criterion) for model selection[12]. In other words, gLASSO contributes to estimation of different models, while EBIC enables selecting the best model. Hence, this technique estimates a model with an extra penalty for model complexity,

therefore jointly performing model-selection and parameter estimation[13]. This helps detect the edges which are most likely true and remove the edges that are likely spurious (accounting for type 1 and type 2 errors).

The network estimation was implemented using the *estimateNetwork* function from the *bootnet* package in R (reference). The estimations were done with default values of parameters, also used in recent relevant studies[14]. Two hyperparameters worth highlighting are *gamma* and *lambda*. The gamma is the tunning parameter used as part of EBIC to define the level to which parsimonious models are preferred. The default gamma value, implemented in the present work, is 0.5 and is typically used for GGM[15]. The lambda parameter is used in GLASSO regularisation and it controls the level of sparsity, i.e., how strong an edge needs to be in order to be kept in the network model. Epskamp and Fried[13] have shown that this approach (EBIC-gLASSO) tends to show high specificity (not estimating edges that are not true) but a varying level of sensitivity (the ability to estimate edges that are present in the true network).

Network structure was visualised using the Fruchterman-Reingold algorithm which aims to place more strongly connected nodes closer together in space and nodes with weaker connections further apart[16]. It is an iterative force-directed algorithm where nodes are initially placed in a circle layout and then in each iteration, each node is repulsed by all other nodes, while node that are more strongly connected are attracted and placed closer to each other. The detection of bridge nodes enabled us to determine the strongest links between domains, i.e., which EF node was most strongly connected to mathematics nodes, and vice versa. Finally, to determine whether there were clusters of nodes in the network, and whether the cluster structure changed with the intervention, we ran cluster analysis. In graph-based approaches, the presence of clusters shows that some nodes are more strongly related than others and it is determined via a data-driven approach.

We also run cluster analysis on the estimated networks. This is a data-driven approach to detect whether nodes in the network are organised into communities, i.e., whether some nodes are more strongly connected than others. The cluster analysis was implemented using a Spinglass algorithm, where the number of clusters and cluster membership are data-driven, rather than predefined. As the outcome of the Spinglass algorithm can be sensitive to the initial random seed, we ran the clustering algorithm 1000 times with a different seed each time, and the most frequent clustering result is reported (the cluster structure was highly reliable, with cluster replicability ranging 78.3-94.6%). Detailed definitions of the network parameters we extracted are presented in Supplementary Table 1, and in Figure 1 in the main manuscript.

Supplementary Table 1. Summary of network indices for the current network analyses

Network Index	Definition
**Overall correlation coefficient**	Computes the overall correlation coefficient between all edges of two networks, to quantify degree of similarity between networks before and after the intervention.
**Centrality: Strength**	Shows how strongly a node is directly connected to other nodes in the network, calculated as a sum of absolute edge weights between the node of interest and all other nodes the node is directly connected to.
**Centrality: Expected Influence**	Considers the sign of the edges as positive or negative, and it is calculated as a sum of edge weights. It converges with strength when all edges in the network are positive.
**Centrality: Closeness**	Shows how strongly a node is indirectly connected to all other nodes in the network, obtained by calculating the inverse of the sum of distances from a node to all other nodes in the network.

**Centrality: Betweenness**	Reveals how often a node connects other nodes, i.e., how often it lies in the shortest path between other nodes.
**Bridge nodes**	Nodes in one group of measures (e.g., EFs or mathematics) that are most strongly connected to all nodes from the other group.
**Clusters of nodes**	Data driven clusters of nodes that are more strongly related than others.

Note. Network indices are summarised here for ease of reference for all readers.

Supplementary Results

Intervention Acceptability and Feasibility

Themes surrounding acceptability were extracted from interviews at the end of intervention delivery. As a whole, multiple benefits were reported for practitioners and children (see Supplementary Table 2). At the same time, staffing issues and adaptability to children with special educational needs (SEN) or English as an additional language (EAL) were reported as the most frequent barrier to the programme.

Supplementary Table 2. Themes emerging from interviews

Theme	Num. Settings	Examples (anonymised by setting)
Benefits of the programme		
Useful activities	5	*[the activities] helped the most when I needed structure and calm in the classroom*<br>*The activities were all fun for the children, and it was interesting to see how much variability there was in the children's performance*
Staff attitudes and behaviour	4	*The ONE helps practitioners to understand where the children's skills are – we are surprised sometimes!*<br>*We were already mathematics focused, but now we have more ideas and more purpose.*
Staff knowledge development and PD	3	*Mathematics has always been a priority in our routine, so there was not a major change. We are thinking more about executive functions. We know that just a few tweaks can make an activity more challenging.*<br>*The training was really interesting and eye-opening – I learnt lots of new things and it has made me observe the children in a new light.*
Children's skill development	3	*The children's confidence improved as well as their mathematics skills*<br>*Challenge became less scary*<br>*It's all to do with the skills they will need when they start school – remembering, counting, sitting still, listening, teaching each other, communication, subitising, shapes, words, preposition words*
Suitable for children with SEN	1	*[SEN child] enjoyed numbers and sequences, number line worked well for her to practice inhibition and gave her a visual aid*
Other benefits for staff	1	*This was a great extra learning opportunity as part of my level 3 EY diploma*

Barriers to taking part		
Staffing issues	6	*The timing was hard because of annual leave and sickness – but it was helpful to have activities that could group children together and adapt to their abilities in one go*
Child ability	4	*Some activities were better than others. The content was not always appropriate for EAL and SEN children.*
Reporting on activities	3	*I didn't love reporting – it was difficult to do right after activity and difficult to remember to do it afterwards*<br>*We struggled with filling in the paperwork. Maybe an electronic version would work better. We already take photos to track the children's progress*
Covid-19 pressures	2	*The children's behaviour has been more challenging since the covid-19 pandemic*
Planning and time	2	*Training sessions should be longer to allow time for planning. A basic problem was that there was no time to communicate and plan within our team across different rooms.*<br>*There are constant changes in our routine, so there's no point trying to plan*
Preschool ethos	1	*The main barrier was the ethos of the preschool. We have no time to plan activities and the children are supposed to spend all day doing free play – there is no time for organised adult-led activities*
Staff capacity and motivation	1	*We have had staff difficulties, with less commitment and buy in from some newer colleagues*<br>*It is difficult to include agency staff in the intervention and core staff have limited time*

Quantitative acceptability indices also came from practitioners' feedback on the play-based activities. The average mean rating per activity was high, 2.7 (SD = 0.26, range = 2.3 – 3.0). Information on adherence to PD and dosage of activities per setting is summarised in Supplementary Table 3. Fidelity was computed using two indicators from the observation sessions. Prior to the observation, the researcher listed five key features of the activity. As the intervention activities were designed to be adaptable to the needs of a specific setting, the observation scheme measured the adherence to the mathematics and EF goals of the activity, and the educator's ability to adapt activities to the children present. Each of these key points were coded (yes/no) depending on whether they had been observed in the activity, and a final percentage score was calculated based on the number of points achieved out of a possible

five. A second indicator of fidelity was calculated using four items on a Likert scale related to adherence to the activity card (e.g., "*The activity contained appropriately challenging executive challenge, as outlined on the activity card.*"). These four items were averaged and a percentage score was calculated per setting. Average fidelity was high, 78.4%, but fidelity was variable across settings (17.1%-100%).

Supplementary Table 3. Adherence to PD, activity dosage and fidelity across settings

Setting	Number of PD sessions attended	Average (SD) number of staff at each session	Proportion of activities completed (out of a possible 3 per week)	Additional activities across the programme	Fidelity Score (%)
A	100%	3.00(0)	100%	3	96.4%
B	100%	2.00(0)	100%	9	81.1%
C	100%	2.50(.500)	100%	5	37.9%
D	100%	5.00 (0)	97.2%	4	100%
E	100%	5.00(0)	86.1%	4	100%
F	100%	5.75(.433)	72.2%	0	96.4%
G	100%	5.75(.829)	63.9%	0	98.2%
H	100%	2.00(0)	25.0%	0	17.1%

Additional Details on Efficacy Findings.

Supplementary Table 4 reports all condition means for the main effects of intervention, EYPP eligibility and their interactions, for all mathematics variables. For EYTN, there was a statistically significant main effect of Intervention group, driven by higher improvements in numeracy for children in the Intervention group (T1 = 28.59; T2 = 35.78) compared to children in the Control group (T1 = 23.06; T2 = 28.92). There were also main effects of intervention on Give N and Number Comparison. EYPP eligibility had a significant main effect on all mathematics variables except for Count High and Order Processing. For all main effects, disadvantaged children (EYPP eligible children) had significantly lower scores compared to non-EYPP eligible children. In addition, for EYTN, there was also a statistically

significant Intervention * EYPP interaction effect. Furthermore, for spatial skills (as indexed by BAS3-PC), there was also an Intervention * EYPP eligibility interaction effect. Supplementary Table 5 reports all condition means for these effects, but for the executive function variables. There was a main effect of intervention on Corsi Blocks Score, but there were no other statistically significant main intervention effects. However, EYPP eligibility had a significant main effect on all EF variables, except for Rabbits and Boats. For all main effects, disadvantaged children (EYPP eligible children) had significantly lower scores compared to EYPP not eligible children and children whose status was unknown. In addition, there were a significant Intervention * EYPP eligibility interaction effect for Corsi Blocks, for Mr Ant and for the EF latent variable, driven by differences of medium size

Supplementary Table 4. Effects of Intervention, Disadvantage and their Interaction on Mathematics variables, with all condition means.

Measure	*Effect*	*Estimated Marginal Means (stdev)*	*F ratio*	*P*
EYTN (raw score)	***Intervention***	M_{Con}=25.99 (14.58); M_{Int}=32.18 (14.70)	**F(1,21)=7.44**	***.012***
	EYPP	M_{EYPP_Yes}=22.61(16.21); M_{EYPP_No}=33.47(13.26); $M_{EYPP\_Unknown}$=31.18(13.21)	**F(2,210)=15.08**	***<.001***
	Int*EYPP	$M_{EYPP\_Yes\_Con}$=16.65 (11.32); $M_{EYPP\_Yes\_Int}$=28.56 (17.42)<br>$M_{EYPP\_No\_Con}$= 32.63 (12.74); $M_{EYPP\_No\_Int}$= 34.32 (13.72)<br>$M_{EYPP\_Unknown\_Con}$= 28.69 (15.17); $M_{EYPP\_Unknown\_Int}$= 33.67 (10.79)	**F(2,210)=3.38**	***.036***
Count High (maximum count)	***Intervention***	M_{Con}=15.53 (14.44); M_{Int}=19.29 (21.09)	F(1,25)=2.00	.170
	EYPP	M_{EYPP_Yes}=13.66(20.24); M_{EYPP_No}=19.44(17.51); $M_{EYPP\_Unknown}$=19.12(21.43)	F(2,164)=2.70	.070
	Int*EYPP	$M_{EYPP\_Yes\_Con}$=11.70 (17.25); $M_{EYPP\_Yes\_Int}$=15.63 (19.77)<br>$M_{EYPP\_No\_Con}$= 17.23 (8.21); $M_{EYPP\_No\_Int}$= 21.66 (22.22)<br>$M_{EYPP\_Unknown\_Con}$= 17.65 (19.54); $M_{EYPP\_Unknown\_Int}$= 20.59 (23.93)	F(2,161)=.03	.967
Give N (score)	***Intervention***	M_{Con}=5.83 (4.79); M_{Int}=8.60 (4.80)	**F(1,27)=11.25**	***.002***
	EYPP	M_{EYPP_Yes}=4.65(4.93); M_{EYPP_No}=8.75(9.17); $M_{EYPP\_Unknown}$=8.24(4.72)	**F(2,282)=20.63**	***<.001***
	Int*EYPP	$M_{EYPP\_Yes\_Con}$=2.68 (4.61); $M_{EYPP\_Yes\_Int}$=6.61 (4.86)<br>$M_{EYPP\_No\_Con}$= 7.72 (4.18); $M_{EYPP\_No\_Int}$= 9.79 (4.19)<br>$M_{EYPP\_Unknown\_Con}$= 7.08 (4.43); $M_{EYPP\_Unknown\_Int}$= 9.40 (5.14)	F(2,365)=.84	.431
Number Comp (proportion correct)	***Intervention***	M_{Con}=.55 (.21); M_{Int}=.62 (.19)	**F(1,32)=4.58**	***.040***
	EYPP	M_{EYPP_Yes}=.55(.19); M_{EYPP_No}=.63(.20); $M_{EYPP\_Unknown}$=.58(.22)	**F(2,216)=4.20**	***.016***
	Int*EYPP	$M_{EYPP\_Yes\_Con}$=.48 (.11); $M_{EYPP\_Yes\_Int}$=.61 (.19)<br>$M_{EYPP\_No\_Con}$= .61 (.21); $M_{EYPP\_No\_Int}$= .65 (.19)<br>$M_{EYPP\_Unknown\_Con}$=.56 (.24); $M_{EYPP\_Unknown\_Int}$=.60 (.21)	F(2,219)=1.32	.270
Number Naming (score)	***Intervention***	M_{Con}=11.47 (5.87); M_{Int}=11.53 (5.97)	F(1,38)=.003	.956
	EYPP	M_{EYPP_Yes}=9.56 (7.11); M_{EYPP_No}=13.27 (4.98); $M_{EYPP\_Unknown}$=11.68 (5.90)	**F(2,276)=9.76**	***<.001***
	Int*EYPP	$M_{EYPP\_Yes\_Con}$=8.49 (6.79); $M_{EYPP\_Yes\_Int}$=10.63 (7.16)<br>$M_{EYPP\_No\_Con}$= 13.17 (5.30); $M_{EYPP\_No\_Int}$= 13.37 (4.72)	F(2,278)=1.98	.140

		$M_{EYPP\_Unknown\_Con}$= 12.77 (5.12); $M_{EYPP\_Unknown\_Int}$= 10.58 (6.24)		
Order Processing (score)	***Intervention***	M_{Con}=1.69 (3.78); M_{Int}=1.98 (3.68)	F(1,27)=.27	.610
	EYPP	M_{EYPP_Yes}=1.44 (3.39); M_{EYPP_No}=2.40 (3.38); $M_{EYPP\_Unknown}$=1.67 (3.56)	F(2,187)=1.92	.149
	Int*EYPP	$M_{EYPP\_Yes\_Con}$=.65 (1.12); $M_{EYPP\_Yes\_Int}$=2.23 (3.93)<br>$M_{EYPP\_No\_Con}$= 2.58 (3.87); $M_{EYPP\_No\_Int}$= 2.25 (3.81)<br>$M_{EYPP\_Unknown\_Con}$= 1.85 (4.11); $M_{EYPP\_Unknown\_Int}$= 1.50 (2.76)	F(2,190)=1.75	.177
BAS – Pattern Construction (t-score)	***Intervention***	M_{Con}=50.29 (11.71); M_{Int}=53.26 (11.09)	F(1,27)=2.99	.095
	EYPP	M_{EYPP_Yes}=47.27(13.07); M_{EYPP_No}=54.24(10.38); $M_{EYPP\_Unknown}$=53.81(10.01)	**F(2,203)=10.23**	***<.001***
	Int*EYPP	$M_{EYPP\_Yes\_Con}$=43.31 (10.19); $M_{EYPP\_Yes\_Int}$=51.23 (13.15)<br>$M_{EYPP\_No\_Con}$= 54.83 (10.90); $M_{EYPP\_No\_Int}$= 53.66 (9.95)<br>$M_{EYPP\_Unknown\_Con}$= 52.72 (10.28); $M_{EYPP\_Unknown\_Int}$= 54.90 (9.91)	**F(2,206)=4.26**	***.015***

Note. Abbreviations: EYPP = Eligible for Early Years Pupil Premium, a UK based index of economic disadvantage, coded as Yes, No or Unknown. Con = control, Int – intervention. Con = control, Int – intervention. Int*EYPP = Interaction effect between Intervention and EYPP eligibility. BAS - PC = British Ability Scale, pattern construction.

Supplementary Table 5. Effects of Intervention, Disadvantage and their Interaction on Executive Functions variables, with all condition means.

Measure		*Estimated Marginal Means (stdev)*	*F ratio*	*P*
Corsi Blocks (score)	***Intervention***	M_{Con}=4.48 (2.64); M_{Int}=5.34 (2.81)	**F(1,28)=4.55**	***.042***
	EYPP	M_{EYPP_Yes}=3.40(2.95); M_{EYPP_No}=5.55(2.55); $M_{EYPP\_Unknown}$=5.84(2.67)	**F(2,224)=19.23**	**<*.001***
	Int*EYPP	$M_{EYPP\_Yes\_Con}$=2.60 (2.13); $M_{EYPP\_Yes\_Int}$=4.19 (3.15)<br>$M_{EYPP\_No\_Con}$= 5.68 (2.63); $M_{EYPP\_No\_Int}$= 5.43 (2.50)<br>$M_{EYPP\_Unknown\_Con}$= 5.17 (2.02); $M_{EYPP\_Unknown\_Int}$= 6.51 (3.22)	**F(2,225)=3.87**	***.022***
Mr Ant (score)	***Intervention***	M_{Con}=1.28 (.77); M_{Int}=1.38 (.77)	F(1,24)=.56	.460
	EYPP	M_{EYPP_Yes}=1.16 (.78); M_{EYPP_No}=1.48 (.75); $M_{EYPP\_Unknown}$=1.36(.73)	**F(2,232)=4.18**	***.016***
	Int*EYPP	$M_{EYPP\_Yes\_Con}$=.90 (.76); $M_{EYPP\_Yes\_Int}$=1.42 (.78)<br>$M_{EYPP\_No\_Con}$= 1.51 (.75); $M_{EYPP\_No\_Int}$= 1.44 (.75)<br>$M_{EYPP\_Unknown\_Con}$= 1.44 (.76); $M_{EYPP\_Unknown\_Int}$= 1.28 (.69)	**F(2,236)=4.51**	***.012***
Rabbits & Boats (post-switch score)	***Intervention***	M_{Con}=4.57 (4.29); M_{Int}=4.54 (4.31)	F(1,28)=.001	.973
	EYPP	M_{EYPP_Yes}=4.11 (4.28); M_{EYPP_No}=5.00 (4.32); $M_{EYPP\_Unknown}$=4.55(4.11)	F(2,267)= 1.08	.340
	Int*EYPP	$M_{EYPP\_Yes\_Con}$=4.22 (4.06); $M_{EYPP\_Yes\_Int}$=4.01 (4.21)<br>$M_{EYPP\_No\_Con}$= 5.17 (4.28); $M_{EYPP\_No\_Int}$= 4.84 (4.30)<br>$M_{EYPP\_Unknown\_Con}$= 4.32 (4.09); $M_{EYPP\_Unknown\_Int}$= 4.79 (4.21)	F(2,269)=.164	.849
Go-nogo (impulse control score)	***Intervention***	M_{Con}=.54 (.20); M_{Int}=.54 (.21)	F(1,24)=.03	.863
	EYPP	M_{EYPP_Yes}=.49(.22); M_{EYPP_No}=.56(.21); $M_{EYPP\_Unknown}$=.58(.20)	**F(2,153)=3.96**	***.021***
	Int*EYPP	$ME_{EYPP\_Yes\_Con}$=.48 (.20); $M_{EYPP\_Yes\_Int}$=.49 (.21)<br>$M_{EYPP\_No\_Con}$= .56 (.21); $M_{EYPP\_No\_Int}$= .56 (.21)<br>$M_{EYPP\_Unknown\_Con}$= .57 (.19); $M_{EYPP\_Unknown\_Int}$= .57 (.23)	F(2,153)=.09	.918

Note. Abbreviations: EYPP = Eligible for Early Years Pupil Premium, a UK based index of economic disadvantage, coded as Yes, No or Unknown. Con = control, Int – intervention. Int*EYPP = Interaction effect between Intervention and EYPP eligibility.

Network analysis

Supplementary Figure 1 depicts Spearman correlation matrices of EF and mathematics tasks before the intervention (Time 1), and in the control and intervention group at Time 2. While network models are estimated on partial Spearman correlation matrices, here we present ordinary correlation coefficients to aid visual comparison of correlations between tasks when we do not control for concurrent correlations with all other tasks.

Following network estimation, we tested accuracy and stability of the estimated networks. This enabled us to determine how dependent the estimated models are on a specific dataset the estimation is based on. We assessed stability of centrality indices by case-dropping bootstrap[13]. This approach involved several steps, starting with estimating centrality indices for the full dataset. Then, we subseted the data by dropping 10% of the dataset and calculate the centrality indices for the reduced dataset, and repeated this step many times (in our case, 2500 times). We repeated this iteratively for reduced datasets where we drop 20%, then 30% etc of the participants. Comparing (by means of correlation coefficients) the original centrality indices (networks estimated on the full dataset) and indices obtained from reduced datasets, speaks to the stability of the estimated networks. As shown in Supplementary Figure 2, the estimated nodes were highly stable, as the correlation coefficients remained higher than 0.75 even when we removed half of the dataset.

In addition to node stability, we interrogated edge accuracy using a non-parametric bootstrapping procedure[13]. After calculating edge weights of the network estimated on the full sample, we generated a dataset from the original dataset by sampling with replacement, and calculated edge weights for this generated dataset, and repeated this 2500 times. Then, we use the computed statistics to draw confidence intervals (Supplementary Figure 3). As pointed out by Epskamp, it is important to note that these confidence intervals cannot be interpreted in a usual way (i.e., that if a confidence interval contains zero, the edge should not

be considered different from zero), because the estimates are biased towards zero due to regularisation is used in network estimation. In addition to the reflection on the main bridge node reported in the main manuscript, a number of bridge nodes emerged in the mathematics cluster. In the mathematics cluster, the EYTN and BAS-PC were identified as the bridge nodes: EYTN was most strongly connected to Mr Ant in the T1 (r = 0.173) and control network (r = 0.173), and to Corsi Blocks in the intervention network (r = 0.335), and it is worth noting that both Mr Ant and Corsi Blocks index maintenance in memory. The BAS-PC node as a bridge node was most strongly connected to Corsi Blocks in the T1 network (r = 0.307) and in the control network (r = 0.251), and to Rabbits & Boats (indexing cognitive flexibility) in the intervention network (r = 0.173).

Finally, data-driven cluster analyses identified three clusters in all three networks (Figure 6, main manuscript) but the structure of clusters (i.e., the nodes which comprise each cluster) differed for the intervention network. The T1 network comprised of three clusters: Cluster 1 consisted of tasks requiring numerical skills alone (Count High, Number Naming and Give N); Cluster 2 of the EF nodes (Corsi Blocks, Go / No-Go, Mr Ant) and BAS-PC (spatial skills); and Cluster 3 contained EYTN, Number Comparison, Order Processing and Rabbits & Boats. The control network had an EF & BAS-PC cluster that was identical to the T1 network, but EYTN and Rabbits & Boats clustered with the numerical tasks (Count High, Number Naming and Give N); and Cluster 3 consisted of Number Comparison and Order processing (Figure 6b). In the intervention network (Figure 6c), there was a more prominent change in cluster structure. While Cluster 1 comprised of the tasks requiring similar numerical skills (Count High, Number Naming and Give N), most EF and mathematics nodes were grouped together in a big cluster (Order Processing, Number Comparison, Rabbits & Boats, Go/No-Go, BAS and Mr Ant). Finally, EYTN and Corsi Blocks formed one central, separate cluster.

Supplementary Figure 1. Correlation patterns **(a)** before the intervention, and after the intervention in the **(b)** control group and **(c)** the intervention group.

The heatmaps depict Spearman correlations (please note that these are not partial correlations).

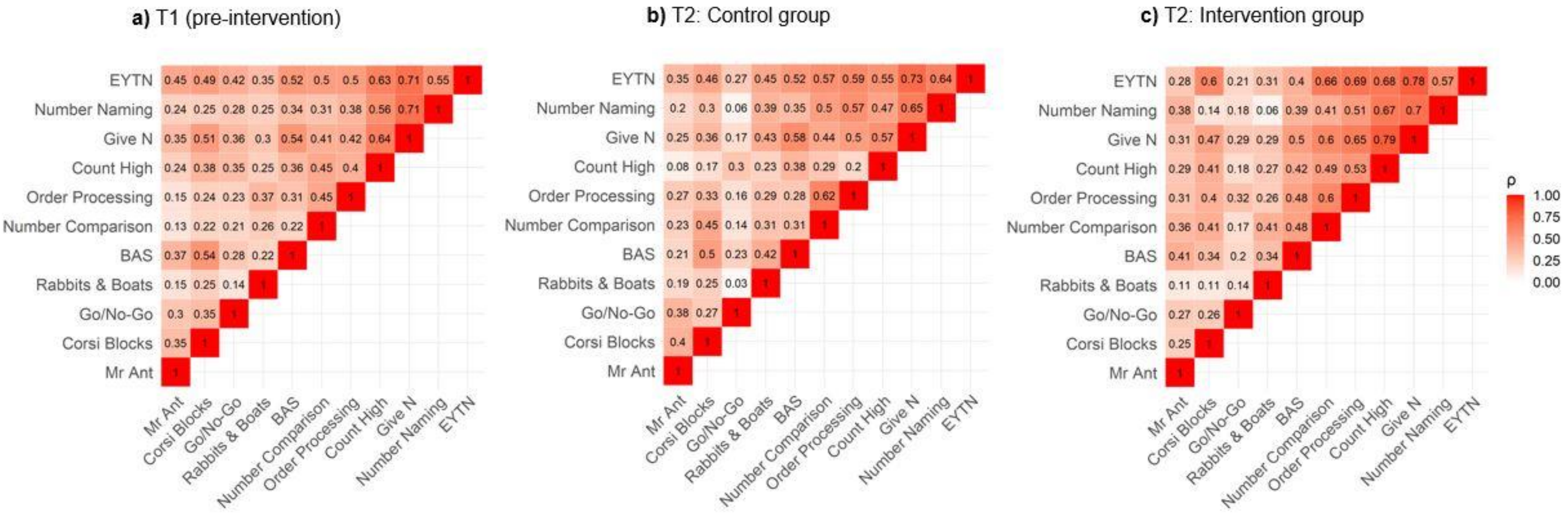

Supplementary Figure 2. Node stability estimates **(a)** before the intervention, and after the intervention in the **(b)** control group and **(c)** the intervention group. The node stability is estimated using a case-dropping subset bootstrap, where the data is subset by dropping an increasing percentage of participants, and for each subset we estimate the centrality measures and access their similarity to the centrality measures of the full dataset (nBoots = 2500).

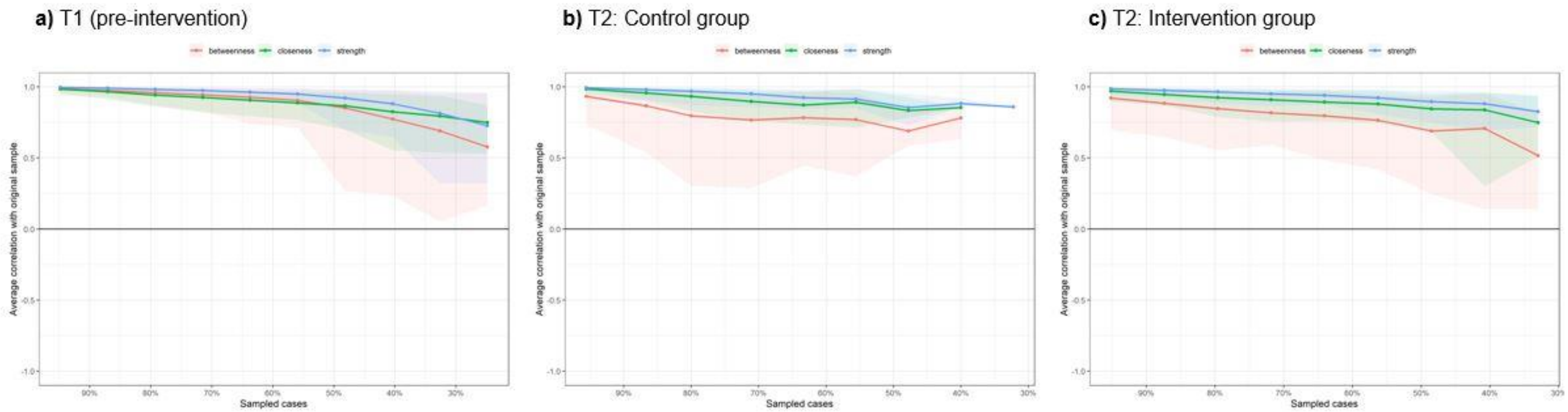

Supplementary Figure 3. Edge stability estimates (nBoots = 2500) for each network edge **(a)** before the intervention, and after the intervention in **(b)** the control group and **(c)** the intervention group. The plots show edge estimate in the sample (depicted in red) and the mean edge estimate in the bootstrapped sample (depicted in black). The grey area shows the 95% confidence interval for the bootstrapped edge weights.

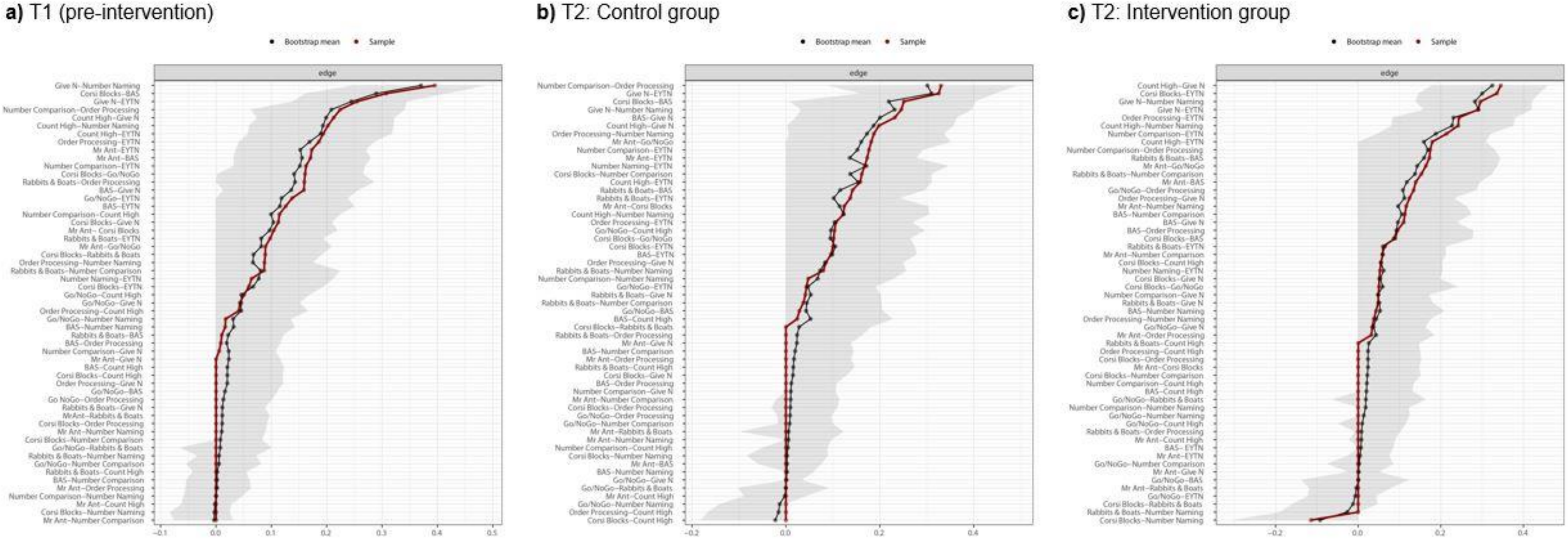

Supplementary References

1 Howard, S. J., Neilsen-Hewett, C., de Rosnay, M., Melhuish, E. C. & Buckley-Walker, K. Validity, reliability and viability of pre-school educators' use of early years toolbox early numeracy. *Australasian Journal of Early Childhood* **47**, 92-106 (2022). https://doi.org/10.1177/18369391211061188

2 Coolen, I. *et al.* Domain-general and domain-specific influences on emerging numerical cognition: Contrasting uni-and bidirectional prediction models. *Cognition* **215** (2021). https://doi.org/10.1016/j.cognition.2021.104816

3 Cahoon, A., Gilmore, C. & Simms, V. Developmental pathways of early numerical skills during the preschool to school transition. *Learning and Instruction* **75** (2021). https://doi.org/10.1016/j.learninstruc.2021.101484

4 Wynn, K. CHILDRENS UNDERSTANDING OF COUNTING. *Cognition* **36**, 155-193 (1990). https://doi.org/10.1016/0010-0277(90)90003-3

5 Batchelor, S., Keeble, S. & Gilmore, C. Magnitude Representations and Counting Skills in Preschool Children. *Mathematical Thinking and Learning* **17**, 116-135 (2015). https://doi.org/10.1080/10986065.2015.1016811

6 Nosworthy, N., Bugden, S., Archibald, L., Evans, B. & Ansari, D. A Two-Minute Paper-and-Pencil Test of Symbolic and Nonsymbolic Numerical Magnitude Processing Explains Variability in Primary School Children's Arithmetic Competence. *Plos One* **8** (2013). https://doi.org/10.1371/journal.pone.0067918

7 Swinson, J. (Taylor & Francis, 2013).

8 Blakey, E. *et al.* The Role of Executive Functions in Socioeconomic Attainment Gaps: Results From a Randomized Controlled Trial. *Child Development* **91**, 1594-1614 (2020). https://doi.org/10.1111/cdev.13358

9 Alloway, T. P. & Passolunghi, M. C. The relationship between working memory, IQ and mathematical skills in children. *Learning and Individual Differences* **21**, 133-137 (2011). https://doi.org/10.1016/j.lindif.2010.09.013

10 Howard, S. J. & Melhuish, E. An Early Years Toolbox for Assessing Early Executive Function, Language, Self-Regulation, and Social Development: Validity, Reliability, and Preliminary Norms. *Journal of Psychoeducational Assessment* **35**, 255-275 (2017). https://doi.org/10.1177/0734282916633009

11 Snijders, T. & Bosker, R. Multilevel Analysis: An Introduction to Basic and Advanced Multilevel Modeling. *http://lst-iiep.iiep-unesco.org/cgi-bin/wwwi32.exe/[in=epidoc1.in]/?t2000=013777/(100)* (1999).

12 Friedman, J., Hastie, T. & Tibshirani, R. Sparse inverse covariance estimation with the graphical lasso. *Biostatistics* **9**, 432-441 (2008). https://doi.org/10.1093/biostatistics/kxm045

13 Epskamp, S. & Fried, E. I. A Tutorial on Regularized Partial Correlation Networks. *Psychological Methods* **23**, 617-634 (2018). https://doi.org/10.1037/met0000167

14 Menu, I., Rezende, G., Le Stanc, L., Borst, G. & Cachia, A. A network analysis of executive functions before and after computerized cognitive training in children and adolescents. *Scientific Reports* **12** (2022). https://doi.org/10.1038/s41598-022-17695-x

15 Foygel, R. & Drton, M. Extended Bayesian information criteria for Gaussian graphical models. *Advances in neural information processing systems* **23** (2010).

16 Fruchterman, T. M. & Reingold, E. M. Graph drawing by force-directed placement. *Software: Practice and experience* **21**, 1129-1164 (1991).
